# Isoliquiritin ameliorates depression by suppressing NLRP3-mediated pyroptosis via miRNA-27a/SYK/NF-κB axis

**DOI:** 10.1186/s12974-020-02040-8

**Published:** 2021-01-05

**Authors:** Yuanjie Li, Wen Song, Yue Tong, Xia Zhang, Jianjun Zhao, Xiaojuan Gao, Jingjiao Yong, Hanqing Wang

**Affiliations:** 1 College of Pharmacy, Ningxia Medical University, 1160 Shengli Street, Yinchuan, 750004 Ningxia People’s Republic of China; 2 Ningxia Engineering and Technology Research Center for Modernization of Regional Characteristic Traditional Chinese Medicine, Ningxia Medical University, Yinchuan, People’s Republic of China; 3 Key Laboratory of Hui Ethnic Medicine Modernisation, Ministry of Education, Ningxia Medical University, Yinchuan, People’s Republic of China

**Keywords:** Depression, miRNA-27a, NLRP3, Pyroptosis, Isoliquiritin

## Abstract

**Background:**

The NLRP3-mediated pyroptosis, which could be regulated by miRNA-27a, is a key player in the development of depression. Isoliquiritin is a phenolic flavonoid compound that has been demonstrated to suppress NLRP3-mediated pyroptosis. However, it is still unknown whether isoliquiritin could confer antidepressant activity via decreasing NLRP3-mediated pyroptosis by stimulating miRNA-27a. Thus, in the current study, we explored the antidepressant activity of isoliquiritin and its underlying mechanism.

**Methods:**

Expression of miRNA-27a in depressed patients or mice was measured using qRT-PCR. Luciferase reporter assay was performed to illustrate the link between miRNA-27a and SYK. Lipopolysaccharide (LPS) and chronic social defeat stress (CSDS) depression models were established to investigate the antidepressant actions of isoliquiritin. Changes in miRNA-27a/SYK/NF-κB axis and NLRP3-mediated pyroptosis were also examined. The role of miRNA-27a in isoliquiritin-related antidepressant effect was further investigated by using miRNA-27a inhibitors and mimics of miRNA-27a.

**Results:**

Our results showed the miRNA-27a expression was downregulated in the serum of depressed patients, and decreased serum and hippocampus expression of miRNA-27a were observed in rodent models of depression. SYK gene expression was significantly reduced by miRNA-27a mimic incubation. Isoliquiritin profoundly attenuated LPS or CSDS-induced depressive symptoms, as well as CSDS-induced anxiety behavior. In the hippocampus, LPS and CSDS decreased miRNA-27a mRNA expression; increased the protein levels of SYK, p-NF-κB, and NLRP3: cleaved Caspase-1, IL-1β, and GSDMD-N: and elevated the concentration of IL-1β, IL-6, and TNF-α, which were all restored by isoliquiritin administration. Meanwhile, isoliquiritin upregulated the hippocampal NeuN protein level, improved the survival and morphology of neurons, and decreased pyroptosis-related neuronal cell death. Moreover, isoliquiritin protected primary microglia against LPS and adenosine triphosphate (ATP) elicited NLRP3 inflammasome activation in vitro, evidenced by declined protein levels of p-NF-κB, NLRP3; cleaved Caspase-1, IL-1β, and GSDMD-N; upregulated miRNA-27a mRNA expression; and decreased the mRNA and protein levels of SYK. Nevertheless, miRNA-27a inhibitors significantly reversed isoliquiritin-generated therapeutic efficacy in CSDS mice and in vitro. Furthermore, the cytoprotective effect of isoliquiritin was similar to that of miRNA-27a mimics in LPS and ATP-treated primary microglia.

Taken together, these findings suggest that isoliquiritin possesses potent antidepressant property, which requires miRNA-27a/SYK/NF-κB axis controlled decrease of pyroptosis via NLRP3 cascade.

**Supplementary Information:**

The online version contains supplementary material available at 10.1186/s12974-020-02040-8.

## Background

Depression is a pervasive and debilitating psychiatric disorder that is characterized by low mood, loss of pleasure, social avoidance, etc. It affects approximately 322 million people worldwide, leading to a major social and economic burden [1]. Although multiple antidepressants are available for depression treatment, first-line drugs, including selective serotonin reuptake inhibitors (SSRIs), exhibit low curative ratio, extensive side effects, and poor treatment compliance. Hence, novel therapies with higher efficacy and fewer adverse effects are urgently needed [2].

Current evidence demonstrates that the development of depression is associated with NLRP3-mediated pyroptosis. Pyroptosis is a Caspase-1-dependent programmed cell death. In contrast to apoptosis, the process of pyroptosis is proinflammatory and initiated by inflammasomes. NLRP3 is a typical representative of NOD-like receptors (NLRs), which play essential roles in triggering inflammasome-mediated neuroinflammation in microglia. Activated NLRP3 induces the cleavage of pro-Caspase-1 into activated Caspase-1, which drives the maturation of proinflammatory cytokine IL-1β and pyroptosis executor GSDMD, consequently leading to pyroptosis and the production of proinflammatory factors such as IL-6 and TNF-α [3]. Clinical evidence demonstrated that the concentrations of NLRP3 activated cytokines such as IL-1β, IL-6, and TNF-α were elevated in the cerebral spinal fluid (CSF) and serum of patients with depression [4]. Therapy contributing to the decrease of NLRP3-regulated pyroptosis was found to exert significant antidepressant-like actions [5].

MicroRNAs (miRNAs) are a class of small, single-stranded, non-coding RNAs that negatively regulate target gene transcription via binding to the partial sequence homology of the 3′-untranslated region of their target mRNAs [6]. As one of hundreds of microRNAs, miRNA-27a has been reported to control the NLRP3 cascade via SYK signaling [7, 8]. Spleen tyrosine kinase (SYK) is a cytoplasmic protein-tyrosine kinase that expresses in either hematopoietic or epithelial cells. It is responsible for a variety of physiological function, such as cellular adhesion and immune recognition [9]. The activation of miRNA-27a could downregulate SYK expression, which then stimulates NF-κB and facilitates NLRP3 signal [10].

Isoliquiritin is one of the major flavonoid glycoside compounds extracted from *Glycyrrhiza uralensis*, which possess broad spectrum of pharmacological properties, for instance, antiangiogenic, anti-neurotoxic, and anti-tumor [11–13]. Isoliquiritin has also been reported to exert antidepressant-like actions. The immobility time in tail suspension test (TST) and forced swimming test (FST) was significantly reduced after treatment with isoliquiritin, reflecting its impact on alleviating behavioral despair [14]. In addition, isoliquiritin is capable of deactivating NF-κB regulated inflammation response. Decreased mRNA levels of NF-κB and IL-6 were detected in hepatocytes after isoliquiritin intervention [15]. In the kidney of membranous glomerulonephritis rats, isoliquiritin significantly downregulated the protein expression of NF-κB, as well as the mRNA expressions of NF-κB and IL-1β [16]. These findings lead us to hypothesize that isoliquiritin could ameliorate depression via the miRNA-27a/SYK/NF-κB axis-mediated attenuation of pyroptosis by targeting NLRP3 pathway.

To investigate this hypothesis, first we verified the involvement of miRNA-27a in depression using hippocampus and serum samples obtained from depressed patients or rodent models. The relationship between miRNA-27a and SYK was detected employing luciferase reporter assay. Lipopolysaccharide and chronic social defeat stress models of depression were established to examine the impact of isoliquiritin on depressive behaviors, hippocampal miRNA-27a/SYK/NF-κB cascade and NLRP3-regulated pyroptosis. Finally, the role of miRNA-27a in isoliquiritin-related antidepressant effects was further evaluated using miRNA-27a inhibitors and mimics.

## Materials and methods

### Participants

Adult patients (men 12, women 12) aged 18–60 years were enrolled at Taizhou Fourth People’s Hospital. Patients were diagnosed with MDD according to the *Diagnostic and Statistical Manual of Mental Disorders, 4th ed.-Text Revision (DSM-IV-TR)*. The severity of MDD was assessed using the 17-item Hamilton Depression Rating Scale (HAMD-17). Eligible patients had HDRS-17 total scores ≥ 18 at study entry and had stopped taken anti-depressive or other psychiatric medications for more than 3 weeks. Patients pregnant or with a primary diagnosis of schizophrenia or other psychotic disorders, substance abuse, severe cognitive impairment, or epilepsy were excluded from the study. Healthy control subjects (men 12, women 12) had a HAMD-17 score ≤ 7 and a Montreal Cognitive Assessment (MoCA) score ≥ 26 at study entry. The healthy control group consisted of individuals with no present and past history of psychiatric disorders. Written informed consent was obtained from all participants.

### Animal

Male C57BL6/J mice (4–6 weeks of age) and retired CD-1 mice breeders (≥ 12 weeks of age) were obtained from Shanghai Sipper-BK laboratory animal Co. Ltd (Shanghai, China). Mice were housed in a regulated environment (23 ± 1 °C, 60 ± 5% humidity) with a 12-h light/12-h dark cycle (lights on at 07:00). Animals were allowed to habituate for 1 week before the start of experiments. All studies were conducted in accordance with the Provision and General Recommendation of Chinese Experimental Animals Administration Legislation.

### Drugs

Isoliquiritin (purity ≥ 98%) was obtained from Chengdu Must Bio-Technology Co., Ltd (Sichuan, China). Fluoxetine (Flu) was purchased from Sigma-Aldrich (St. Louis, USA). Flu (20 mg/kg) was selected as positive control based on previous studies [17, 18].

### Experimental design

#### Experiment I: involvement of miRNA-27a expression in depression

qRT-PCR was performed to measure the miRNA expression in the serum and hippocampus samples of depressed patients (*n* = 24 per group) or mice (*n* = 10 per group). Human serum samples were obtained from depression patients and healthy donors at Taizhou Fourth People’s Hospital. The hippocampus and serum samples of mice experienced LPS or chronic social defeat stress (CSDS) challenge were collected.

#### Experiment II: effect of isoliquiritin on LPS-induced depression

Male C57BL6/J mice were randomized into five groups (*n* = 10 per group): control group (vehicle treatment and ICV injection of saline), LPS group (vehicle treatment and ICV injection of LPS), isoliquiritin (10 mg/kg) + LPS group, isoliquiritin (30 mg/kg) + LPS group, and fluoxetine (20 mg/kg) + LPS group. Mice were orally administered with isoliquiritin, fluoxetine, or vehicle (0.9% saline) by gavage daily (8 a.m.) for 14 days. LPS modeling was performed on day 11–12 (9 a.m.). On the 13th day, the sucrose preference test (SPT) (9 a.m.) and TST (3 p.m.) were carried out, and the FST (9 a.m.) was conducted on the 14th day. Subsequently (11 a.m.), mice were anesthetized with 1% pentobarbital sodium, and brains were immediately removed and hippocampus was collected and stored at − 80 °C until further use.

#### Experiment III: effect of isoliquiritin on CSDS-induced depression

Mice were randomly divided into five groups (*n* = 10 per group): control group (vehicle treatment), CSDS group (vehicle treatment), CSDS + isoliquiritin (10 mg/kg) group, CSDS + isoliquiritin (30 mg/kg) group, and CSDS + fluoxetine (20 mg/kg) group. Mice were orally administered with isoliquiritin, fluoxetine or vehicle by gavage daily (8 a.m.) for 14 days. The CSDS modeling was performed from at 9 a.m. from day 1 to 12. On the 13th day, the SPT (9 a.m.), open field test (OFT) (2 p.m.), and TST (5 p.m.) were carried out, and the FST (9 a.m.) and social interaction test (SIT) (3 p.m.) were conducted on the 14th day. Subsequently (5 p.m.), mice were anesthetized with 1% sodium pentobarbital, and brains were immediately removed and hippocampus was collected and stored at − 80 °C until further use.

#### Experiment IV: role of miRNA-27a in isoliquiritin-related antidepressant efficacy

Mice were randomized into five groups (*n* = 10 per group): control + LV-NC group (vehicle treatment), CSDS + LV-NC group (vehicle treatment), CSDS + isoliquiritin (30 mg/kg) + LV-NC group, CSDS + LV-miRNA-27a group (vehicle treatment), and CSDS + isoliquiritin (30 mg/kg) + LVI-miRNA-27a group. Mice were orally administered with isoliquiritin or vehicle by gavage daily (8 a.m.) for 14 days, and lentivirus vectors (Fanyida Biosciences, Nanjing, China) expressing miRNA-27a inhibitor (forward 5′-GGATCCAAGTGTCAGTATTCAAGGCGAATTAAGTGTCATTCAAGGCGGAATTC-3′ and reverse 5′-GAATTCCGCCTTGAATGACACTTAATTCGC CTTGAATA CTGACACTTGGATCC-3′) or control vectors (LV-NC) at a dose of 1 × 10^7^ transducing units each time was i.c.v. injected 1 h prior to drug administration. The CSDS modeling was performed from at 9 a.m. from day 1 to 12. On the 13th day, the SPT (9 a.m.) and TST (3 p.m.) were carried out, and the FST (9 a.m.) and SIT (3 p.m.) were conducted on the 14th day. Subsequently (5 p.m.), mice were anesthetized with 1% sodium pentobarbital, and brains were immediately removed and hippocampus was collected and stored at − 80 °C until further use.

### Lipopolysaccharide (LPS) model

The LPS modeling was performed according to the method reported previously [19]. Briefly, Mice were anesthetized with pentobarbital sodium (80 mg/kg, i.p.) and restrained onto a stereotaxic apparatus. A skin incision was made to expose the skull, and a small burr hole was drilled perpendicularly to the skull. Using an infusion pump, 1 μl saline or 1 μl of 10 mg/ml LPS (L2630; serotype O111:B4; Sigma) in saline was ICV administrated (flow rate, 0.3 μl/min) to mice stereotactically via the coordinates: − 2.5 mm dorsal/ventral, − 1.0 mm lateral, and − 0.5 mm anterior/posterior from bregma. The needle remained in place for 5 min for proper dispersal of the drug.

### Chronic social defeat stress (CSDS)

The CSDS paradigm was conducted as previous described with minor modifications [20]. Eight-week-old Kunming mice were considered “intruder” mice and experienced 12 consecutive days of stress. Briefly, intruder was defeated by a CD-1 mouse. The defeat episode lasted for 5 min, after which the defeated mouse was subjected to continuous psychological stress from a CD-1 mouse through a clear perforated divider allowing for visual, olfactory, and auditory contact in a shared home cage for the next 24 h.

### Behavioral studies

The behavioral tests were performed by the investigators blinded to the study groups.

#### Tail suspension test (TST)

The TST was performed as previously described with minor modifications [19]. The mouse with a medical tape placed 1 cm from the tip of the tail was suspended upside-down for 6 min on the TST instrument holder. The immobility time for each mouse throughout the last 4 min was statistically analyzed by ANY-MAZE software.

#### Forced swimming test (FST)

The FST was performed as previously described with minor modifications [19]. Mice were placed in a transparent cylinder (diameter 10 cm, height 30 cm), containing 20 cm of water at 24 ± 1 °C. The total duration of immobility during the last 4 min of the 6-min session was analyzed.

#### Sucrose preference test (SPT)

The SPT followed a published procedure with minor modifications [21]. Mice were accustomed to 2% sucrose water for 3 consecutive days prior to the test. On the testing day, each mouse was water-deprived for 24 h, and given two drinking bottles: one containing 2% sucrose water and the other regular water. After 2 h, the bottles were weighed and sucrose preference was analyzed according the following formula: sucrose preference = sucrose intake/total water consumption × 100%.

#### Open field test (OFT)

The OFT was applied to evaluate anxiety and locomotor activity in rodents as previously described [21]. Animals were gently placed into open-field chambers (30 × 30 cm) which were equipped with video cameras. During the test, mice were allowed explore the arena for 5 min. Total distance traveled and time spent in the central area automatically were recorded.

#### Social interaction test (SIT)

The social interaction test was used to determine social avoidance behavior [22]. Briefly, the mouse was introduced to an open field chamber where a male CD-1 mouse was present in the mesh cage at one end. Each mouse was allowed to freely explore the environment for 2.5 min with its movement tracked. The mouse was habituated to the chamber in the absence of a CD-1 mouse for 2.5 min prior to the test. Social interaction ratio was calculated according the following formula: Social interaction ratio = time spent in the interaction zone in the presence of target/time spent in the interaction zone in the absence of target × 100%.

### Cell culture and drug treatments

Mouse primary microglia were obtained from the hippocampus of 1-day-old neonatal C57/B6J mice following a published protocol [23]. Briefly, hippocampal tissues were dissected and digested in 0.125% Trypsin-EDTA for 15 min at 37 °C, followed by mechanical shearing. After centrifugation (1000 rpm, 10 min), cells were resuspended in Dulbecco’s modified Eagle medium (DMEM) with additional 10% FBS. Then, the resulting suspension was filtered through a 70-μm filter and cultivated in DMEM supplemented with 10% FBS, 40 U/mL penicillin, and 40 μg/mL streptomycin. Medium was refreshed every 3–4 days. Upon reaching confluence (10–12 days), the microglial cells were separated from the underlying astrocytic monolayer via shaking off for 5 h at 100 rpm. The cells were verified by Iba-1 immunostaining. After 12 h of culture, the microglial cells were ready for use.

Microglial cells were exposed to different concentrations of isoliquiritin for 2 h, and then added with LPS (10 μg/L) and ATP (5 mM) for 12 h to induce NLRP3 inflammasome activation phenotypes [5].

### Quantitative real-time polymerase chain reaction (qRT-PCR) analysis

Total RNA in hippocampus, serum, and cells were extracted with TRIzol reagent (Invitrogen USA) and reverse transcribed with a first strand cDNA synthesis kit (Transgen Biotech, China) following the manufacturer’s guides. GAPDH was used as normalization control for mRNA. The sequences for primers were as follows:

**Table:** 

Primers	Forward	Reverse
miR-27a	5′-TTCACAGTGGCTAAG-3′	5′-GTGCAGGGTCCGAGGT-3′
SYK	5′-CCAACCACCTGACCTACTTTTT-3′	5′-ATTAAGTTCCCTCTCGATGGTG-3′
GAPDH	5′-ATGGAGAAGGCTGGGGCTC-3′	5′-AAGTTGTCATGGATGACCTTG-3

### Luciferase reporter assay

The luciferase reporter assay was performed as previously reported [7]. The HZ0471 luciferase reporter plasmid containing the wild-type (WT) or mutant (MUT) 3′UTR fragment of SYK was co-transfected into HEK293T cells co-transfected with miRNA-27a. After 48 h of cell transfection, the luciferase reporter gene activity was measured using Glomax 2020 luminometer.

### Western blot

The Western blot followed a published procedure with minor modifications [23]. Proteins were extracted from the hippocampus of LPS and CSDS mice, and the treated cells, and total protein concentrations were measured using the Bradford assay kit. Proteins were separated on 10% SDS-PAGE gels and then transferred to polyvinylidene difluoride (PVDF) membranes. After blocking with 3% BSA, the membranes were incubated with primary antibodies at 4 °C overnight. Then, appropriate HRP-conjugated secondary antibodies were added and incubated for 1.5 h at room temperature. The blots were visualized using ECL western blotting detection reagents and analyzed by Image J software. Antibodies were obtained from Cell Signal Technology or Santa Cruz Biotechnology.

### Enzyme-linked immunosorbent assay (ELISA)

After behavioral test, hippocampus or serum was harvest from mice. The concentrations of IL-1β, IL-6, and TNF-α were quantified using ELISA kits (Boster Biological Technology, Wuhan, China) according to the manufacturer’s instructions.

### Nissl staining

Nissl staining was carried out using Nissl Staining Solution (Beyotime Institute of Biotechnology, Shanghai, China) in accordance with the manufacturer’s protocols. Nissl-positive cells were viewed under an optical microscope (Olympus, Japan).

### TUNEL staining

Pyroptosis-related cell death was detected by TUNEL staining using a kit according to the manufacturer's instruction (Roche, South San Francisco, CA, USA).

### MTT assay

The cytotoxicity was analyzed using MTT assay as previously described [24]. The viability of living cells was calculated as percentage of control.

### Immunocytochemistry

The expression of Iba-1 was determined by immunocytochemistry as previously described [23]. Primary microglia cells were fixed with 4% paraformaldehyde for 20 min and then permeabilized with 0.2% Triton X-100 for another 15 min. After blocking with 10% BSA, immunostaining was performed by incubating with rabbit-anti-Iba-1 (2.5ug/ml; Wako 019-19741) overnight at 4 °C followed by goat anti-rabbit IgG H&L (Alexa Fluor® 488, 1: 200) for 2 h at room temperature. The nuclei were stained with DAPI for 10 min. The fluorescent images were examined under a confocal microscope.

### Cell transfection

MiR-27a mimics and inhibitors were transfected with lipofectamine2000 (Life Technologies) following the manufacturer’s protocols. Before transfection, cells were cultured in 6-well plates till they reached 80% confluency. Then, 20 nM miR-27a mimics (same operation for miR-27a inhibitors) were transfected into cells at 37 °C for 24 h using Lipofectamine® 2000 (Invitrogen). miR-27a mimics (5′-CGCCUUGAAUCGGUGACACUU-3′) and miR-27a inhibitors (5′-UGGACAUUUUUAAAAACUGUGAU-3′) were obtained from General Biol Co., Ltd. (Anhui, China).

### Statistical analysis

All data are presented as mean ± SEM. Data were analyzed by unpaired Student’s *t* test for comparison between two groups. Data were analyzed by one-way or two-way ANOVA with Tukey post hoc test for comparison between multiple groups. The criterion for significance was *p* < 0.05.

## Results

### Serum miRNA-27a level is upregulated in MDD patients

**Fig. 1:**
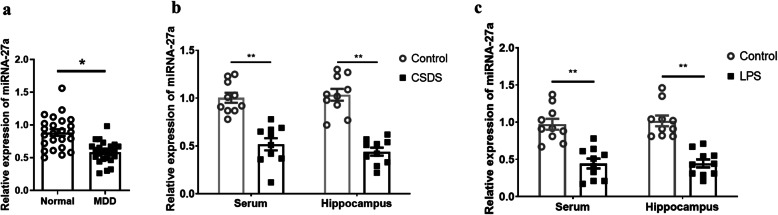
miRNA-27a was downregulated in depression. **a** miRNA-27a mRNA expression in the serum of depressed patients (*n* = 24 per group). **b** miRNA-27a mRNA level in the serum or hippocampus of LPS mice (*n* = 10 per group). **c** miRNA-27a mRNA level in the serum or hippocampus of CDSD mice (*n* = 10 per group). Data are presented as mean ± SEM. *p < 0.05, **p < 0.01, compared to normal control group

To identify the change in miRNA-27a mRNA expression associated with depression, we detected the miRNA-27a mRNA concentration in the serum of depressed patients (Fig. 1a). The qRT-PCR results demonstrated that compared with normal subjects, the miRNA-27a mRNA expression was significantly lower in the serum of depressed patients (*p* = 0.0469). This result indicated that the reduction of miRNA-27a expression was closely associated with the development of depression.

### Serum and hippocampal miRNA-27a expressions were elevated in rodent models of depression

Next, we examined the miRNA-27a expression in rodent models of depression. As illustrated in Fig. 1b and c, LPS (*F* (1, 36) = 68.21, *p* < 0.0001) or CSDS (*F* (1, 36) = 94.51, *p* < 0.0001) challenged mice exhibited decreased miRNA-27a mRNA level in the serum and hippocampus relative to their control counterparts (*p* < 0.05), supporting the reduction of miRNA-27a expression in depression pathology.

### SYK is a target gene of miRNA-27a

**Fig. 2:**
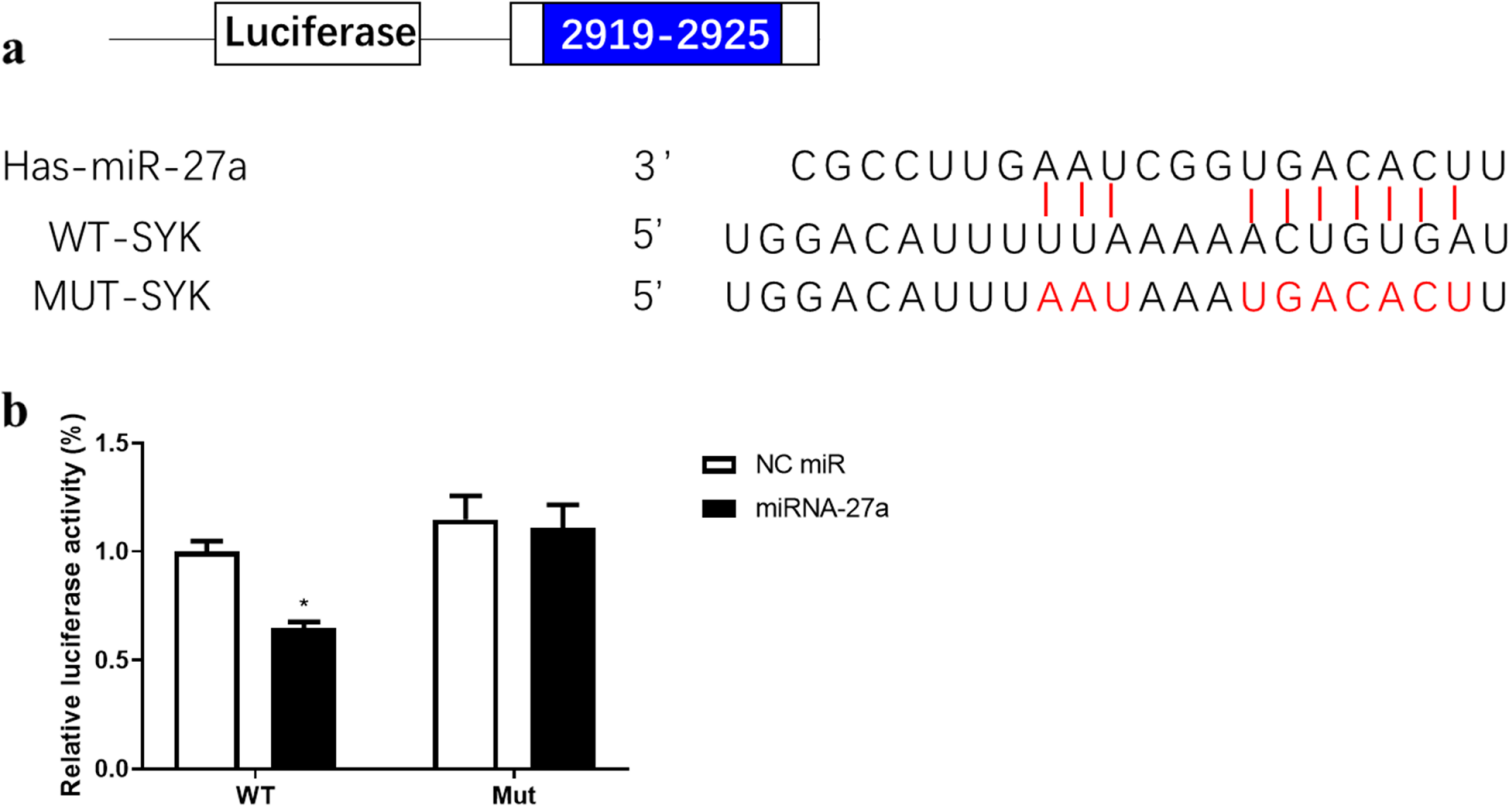
SYK is a target gene of miRNA-27a. **a** miRNA-27a and SYK binding site prediction. **b** Luciferase activity in luciferase reporter gene assay. Data are presented as mean ± SEM (*n* = 4 per group). *p < 0.05, compared to NC miR in WT group

The binding relationship between miRNA-27a and SYK was predicted using online analysis tool TargetScan and verified by employing luciferase reporter assay, in which the 3′-UTR of SYK was fused with a gene encoding luciferase (Fig. 2). The results showed that the relative luciferase activity of HEK293T cells transfected with SYK-WT was notably suppressed by miRNA-27a mimic (*F* (1, 12) = 5.773, *p* = 0.0334), suggesting that SYK serves as a potential target of miRNA-27a.

### Isoliquiritin ameliorated LPS-induced depression

#### Isoliquiritin attenuated LPS-induced depressive behaviors

**Fig. 3:**
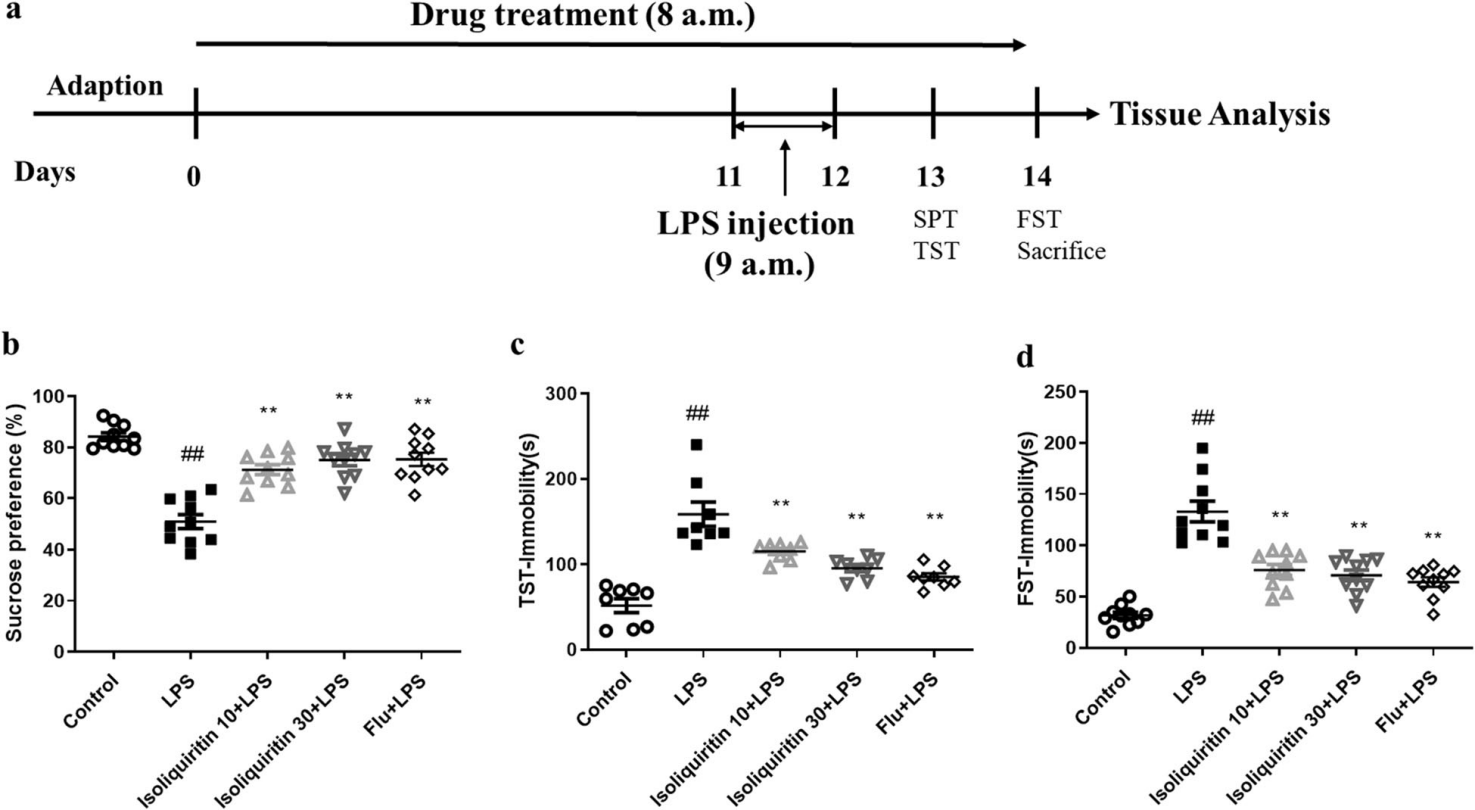
Effects of isoliquiritin on LPS-induced depressive behaviors. **a** Schematic timeline of the experimental procedure. **b** Sucrose preference test. **c** Tail suspension test. **d** Forced swimming test. Data are presented as mean ± SEM (*n* = 8–10 per group). ^##^*p* < 0.01, compared to control group; **p < 0.01, compared to LPS group

We examined whether isoliquiritin treatment could alleviate LPS-induced depressive behaviors in mice (Fig. 3a). Exposure to LPS significantly reduced the sucrose preference ratio (*F* (4, 45) = 29.92, *p* < 0.0001) during the SPT assessment compared with control mice (Fig. 3d). Furthermore, LPS challenge increased the immobility duration in TST (*F* (4, 35) = 25.05, *p* < 0.0001) and FST (*F* (4, 45) = 35.70, *p* < 0.0001) tasks (Fig. 3b, c). Nonetheless, isoliquiritin intervention (10 mg/kg; 30 mg/kg) and Flu completely reversed LPS-induced reduction in sucrose preference and suppressed the immobility time in TST and FST tests. These results indicated that isoliquiritin and Flu were capable of mitigating depressive symptoms in LPS-challenged mice.

#### Isoliquiritin reversed miRNA-27a/SYK/NF-κB cascade

**Fig. 4:**
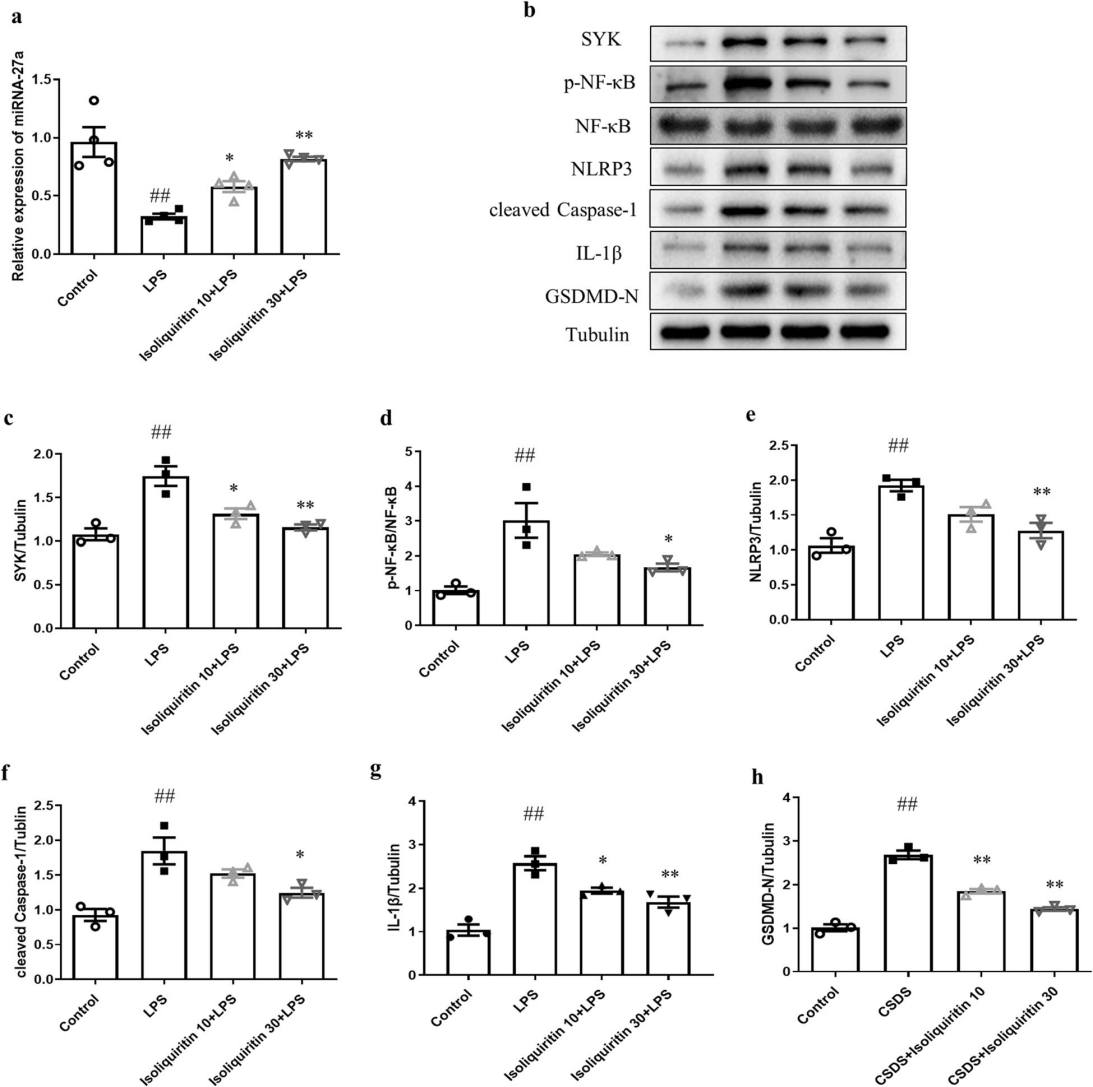
Effects of isoliquiritin on miRNA-27a mRNA expression and protein levels of NLRP3 cascade in the hippocampus of LPS mice. **a** mRNA expression level of miRNA-27a. **b** Representative Western blots. **c**–**h** Protein levels of SYK, p-NF-κB, NLRP3, cleaved Caspase-1, IL-1β, and GSDMD-N. Data are presented as mean ± SEM (*n* = 3–4 per group). ^##^*p* < 0.01, compared to control group; *p < 0.05, **p < 0.01, compared to LPS group

The mRNA level of miRNA-27a in hippocampus was measured by qRT-PCR (Fig. 4a). Compared with the control samples, the miRNA-27a expression was significantly decreased in the hippocampus of LPS mice (*F* (3, 12) = 15.97, *p* = 0.0002). While markedly enhanced miRNA-27a level was observed in LPS mice that received isoliquiritin treatment (10 mg/kg; 30 mg/kg) in comparison to LPS model group. The Western blot assay revealed that LPS modeling elevated SYK (*F* (3, 8) = 16.04, *p* = 0.0010) and p-NF-κB (*F* (3, 8) = 10.22, *p* = 0.0041) protein levels (Fig. 4b–d). However, isoliquiritin intervention had a significant effect on rescuing the expression of SYK (10 mg/kg; 30 mg/kg) and p-NF-κB (30 mg/kg). Collectively, these findings reflected that isoliquiritin administration was effective on rescuing LPS-induced abnormality in the miRNA-27a/SYK/NF-κB cascade.

#### Isoliquiritin prevented LPS-induced inflammation response

**Fig. 5:**
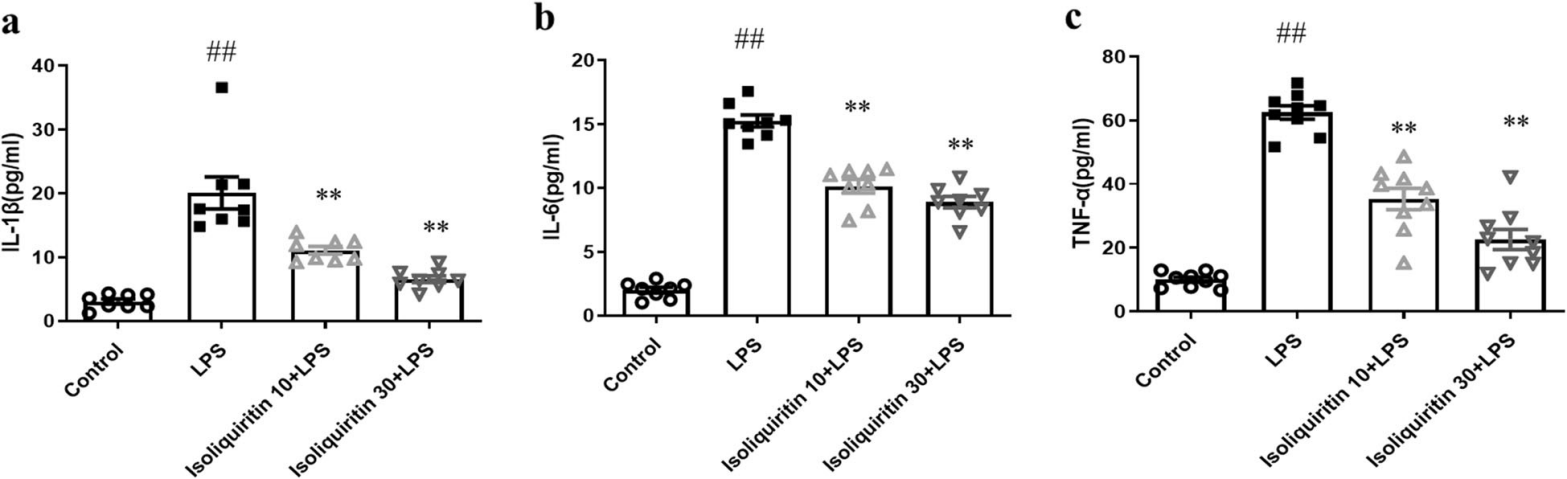
Effects of isoliquiritin on concentration of IL-1β, IL-6, and TNF-α in the hippocampus of LPS mice. **a** IL-1β, **b** IL-6, and **c** TNF-α. Data are presented as mean ± SEM (*n* = 8–9 per group). ^##^*p* < 0.01, compared to control group; **p < 0.01, compared to LPS group

Since the NLRP3 inflammasome signaling is a potential target of NF-κB and serves as an important role in depression, the expression and concentration of the NLRP3 cascade were detected by Western blot and ELISA separately. As presented in Fig. 4e–h, the expression profile of NLRP3 cascade including NLRP3 (*F* (3, 8) = 13.59, *p* = 0.0017), cleaved Caspase-1(*F* (3, 8) = 11.69, *p* = 0.0027), IL-1β (*F* (3, 8) = 25.75, *p* = 0.0002), and GSDMD-N (*F* (3, 8) = 104.4, *p* < 0.0001) were dramatically increased following LPS stimulation, which were all reversed by isoliquiritin 30 mg/kg. In addition, isoliquiritin 10 mg/kg successfully inhibited the upregulation of IL-1β and GSDMD-N. In line with these results, increased serum levels of IL-1β (*F* (3, 28) = 30.24, *p* < 0.0001), IL-6 (*F* (3, 28) = 156.6, *p* < 0.0001), and TNF-α (*F* (3, 32) = 77.39, *p* < 0.0001) induced by LPS challenge were remarkably suppressed by the two dosages of isoliquiritin (Fig. 5).

#### Isoliquiritin affected neuronal cell death

In order to investigate the impact of isoliquiritin on neuronal cell death resulted from pyroptotic cascades, we observed the survival and alternation of neuronal cells, DNA fragmentation and expression level of NeuN using Nissl staining, TUNEL assessment, or Western blot approach.

**Fig. 6:**
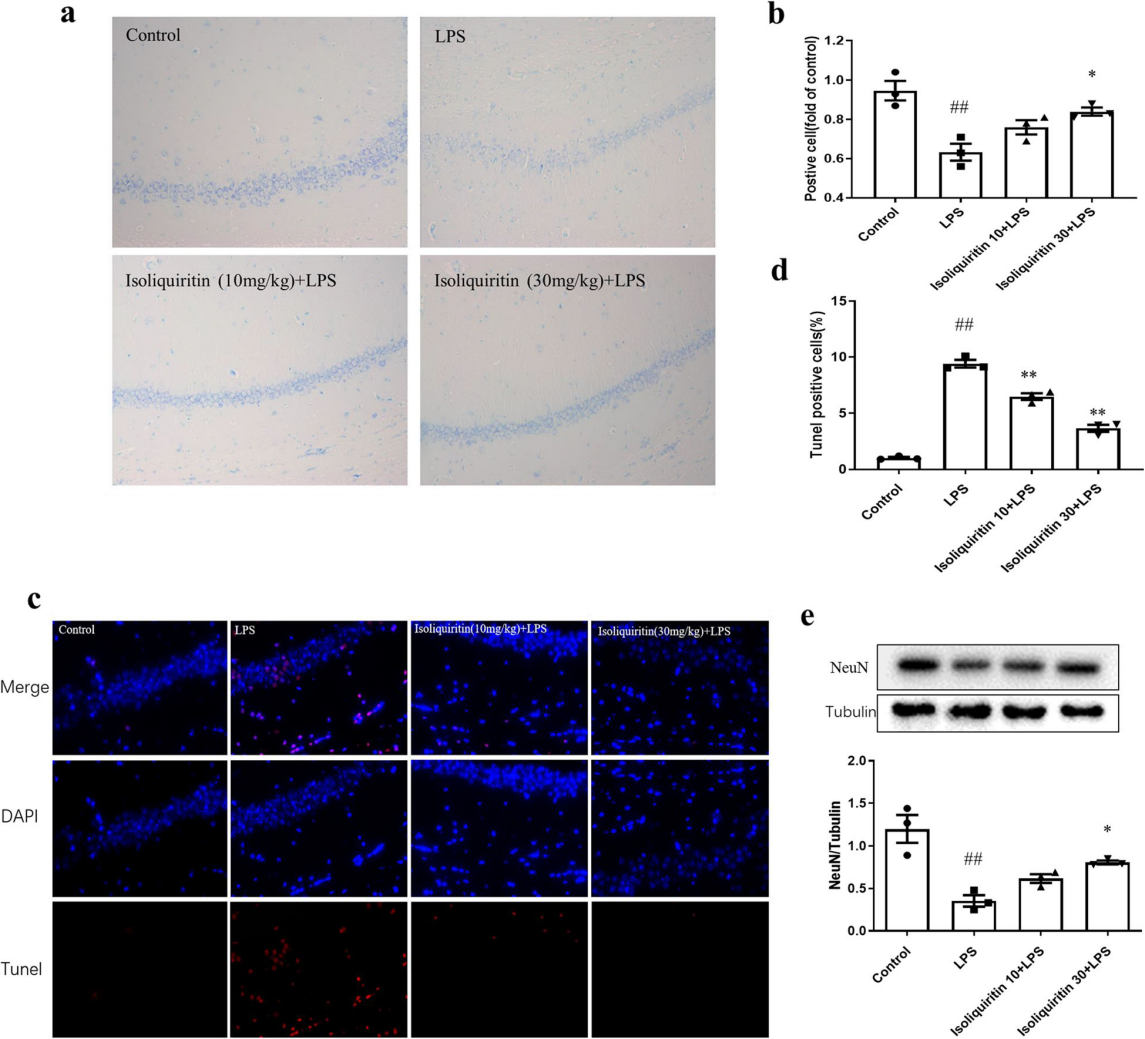
Influence of isoliquiritin on pyroptosis-related neuronal death in the hippocampus of LPS mice. **a**, **b** Nissl staining of survived neurons. **c**, **d** TUNEL assay of neuron death. **e** NeuN protein expression in Western blot. Data are presented as mean ± SEM (*n* = 3 per group). ^##^*p* < 0.01, compared to control group; *p < 0.05, **p < 0.01, compared to LPS group

Nissl staining revealed that the neurons in the hippocampus of control mice were clear and intact. Whereas, LPS mice exhibited reduced survived neurons and increased impaired neurons (*F* (3, 8) = 11.47, *p* = 0.0029), which had irregular neuronal cell bodies, shrinking and hyperchromatic nuclei (Fig. 6a, b). After isoliquiritin administration, the degree of Nissl body loss (30 mg/kg) was attenuated, and the disordered arrangement of neuronal cells was improved.

In TUNEL assay, we observed elevated TUNEL-positive cells in the hippocampus of LPS-challenged mice compared to control group (*F* (3, 8) = 168.2, *p* < 0.0001), which was successfully suppressed by 10 mg/kg or 30 mg/kg of isoliquiritin administration (Fig. 6c, d).

Similar result was observed in Fig. 6e, wherein we present the protein level of NeuN (*F* (3, 8) = 14.93, *p* = 0.0012). Evident enhancement of NeuN protein level was found in isoliquiritin group (30 mg/kg) relative to the LPS group.

Taken together, these findings demonstrated that isoliquiritin protect LPS mice against pyroptotic cascade caused neuronal cell death.

### Isoliquiritin ameliorated CSDS-induced depression

#### Isoliquiritin improved CSDS-induced depression- and anxiety-related behaviors

**Fig. 7:**
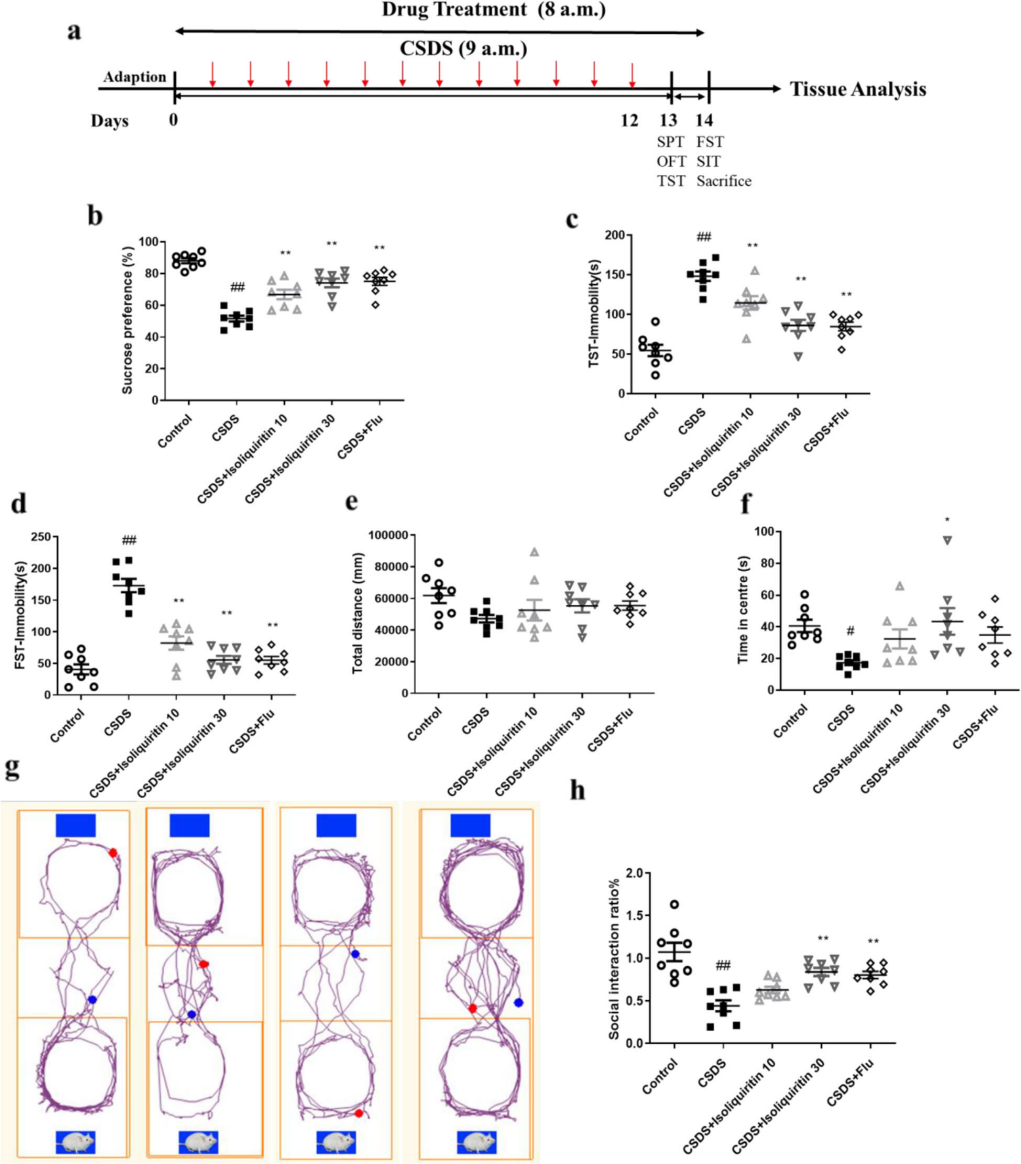
Effects of isoliquiritin on CSDS-induced depression- and anxiety-related behaviors. **a** Schematic timeline of the experimental procedure, **b** sucrose preference test, **c** tail suspension test, **d** forced swimming test, **e**, **f** open field test, and **g**, **h** social interaction test. Data are presented as mean ± SEM (*n* = 8 per group). ^#^*p* < 0.05, ^##^*p* < 0.01, compared to control group; *p < 0.05, **p < 0.01, compared to CSDS group

Depression-like behaviors were examined via multiple behavioral tests, namely SPT, TST, FST, and SIT (Fig. 7a). The model group demonstrated decreased sucrose preference rate in SPT (*F* (4, 35) = 30.97, *p* < 0.0001) as well as extended immobility in TST (*F* (4, 35) = 26.28, *p* < 0.0001) and FST (*F* (4, 35) = 39.49, *p* < 0.0001) tasks when compared with those in the control group (Fig. 7b–d). Treatment with isoliquiritin (10 mg/kg; 30 mg/kg) and Flu significantly increased the sucrose consumption and decreased the immobility time in TST and FST. In addition, after repeated social defeat stress, defeated mice displayed reduced social interaction ratio compared with undefeated controls (*F* (4, 35) = 13.29, *p* < 0.0001), suggesting a development of social avoidance (Fig. 7g, h). Nonetheless, isoliquiritin (30 mg/kg) and Flu administration promoted the social interaction ratio. These findings reflected that isoliquiritin could improve depression-like behaviors in CSDS-induced mice.

OFT was applied to assess locomotor activity and anxiety-like behavior. As presented in Fig. 7e and f, no significant difference was detected in total distance, indicating no alternation of locomotor activity after CSDS modeling or drug treatment. However, CSDS mice exhibited noticeable anxiety-like behavior, as indicated by less time spent in center zone relative to the controls, while this reduction was completely reversed by the administration of isoliquiritin (30 mg/kg) but not Flu, demonstrating that isoliquiritin was efficacious on ameliorating CSDS-elicited anxiety.

#### Isoliquiritin stimulated miRNA-27a/SYK/NF-κB cascade

**Fig. 8:**
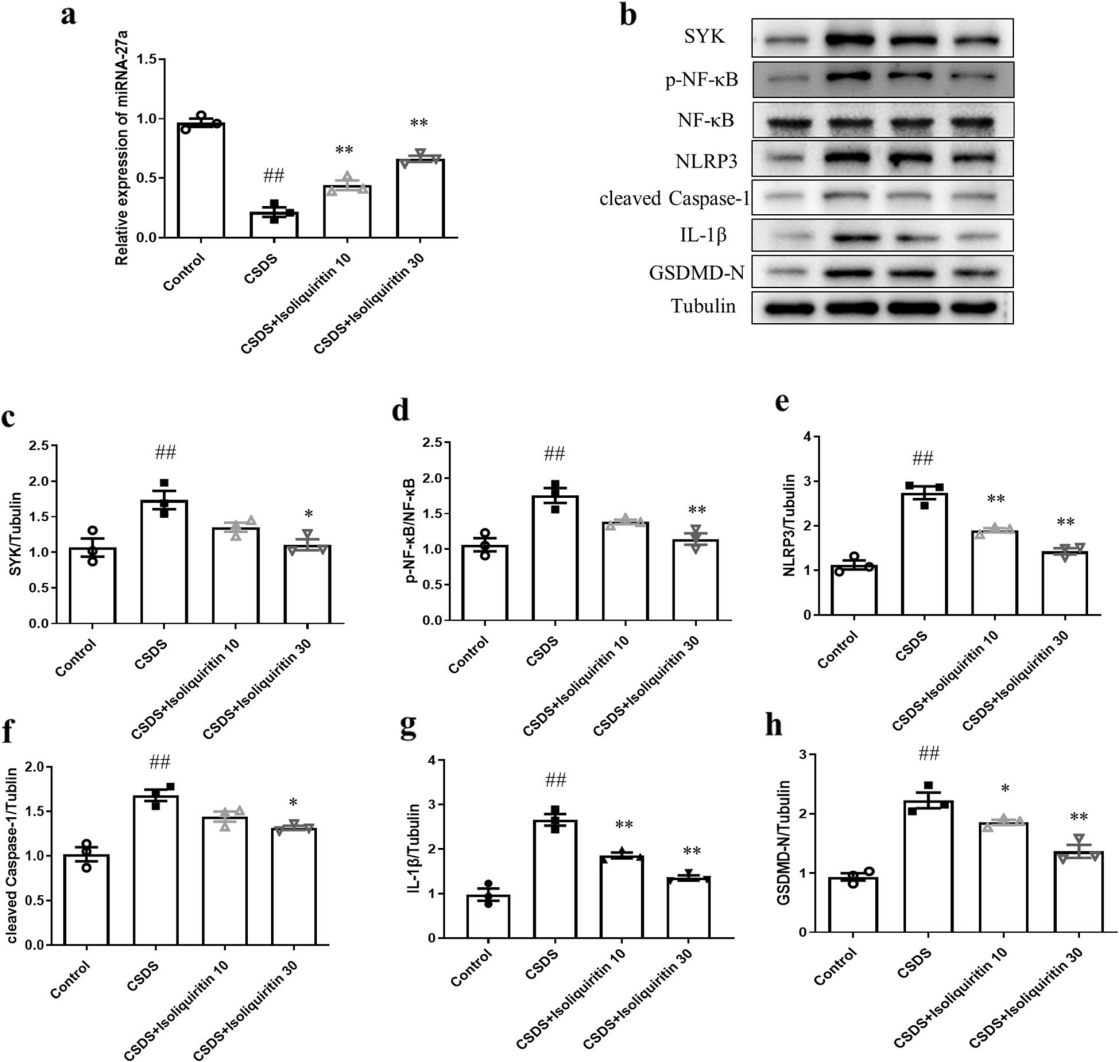
Effects of isoliquiritin on miRNA-27a mRNA expression and protein levels of NLRP3 cascade in the hippocampus of CSDS mice. **a** mRNA expression level of miRNA-27a. **b** Representative Western blots. **c**–**h** Protein levels of SYK, p-NF-κB, NLRP3, cleaved Caspase-1, IL-1β, and GSDMD-N. Data are presented as mean ± SEM (*n* = 3 per group). ^##^*p* < 0.01, compared to control group; *p < 0.05, **p < 0.01, compared to CSDS group

The efficacy of isoliquiritin on the miRNA-27a/SYK/NF-κB cascade in CSDS mice was shown in Fig. 8a–d. After CSDS, the mRNA expression of miRNA-27a (*F* (3, 8) = 79.43, *p* < 0.0001) was decreased in the hippocampus, along with increased protein levels of SYK (*F* (3, 8) = 8.720, *p* = 0.0067) and p-NF-κB (*F* (3, 8) = 14.24, *p* = 0.0014) compared to the control. However, isoliquiritin significantly upregulated the miRNA-27a mRNA level (10 mg/kg; 30 mg/kg) and downregulated the protein expression of SYK (30 mg/kg) and NF-κB (30 mg/kg), indicating that isoliquiritin alleviated miRNA-27a/SYK/NF-κB cascade defects in CSDS mice.

#### Isoliquiritin reduced CSDS-induced inflammation response

**Fig. 9:**
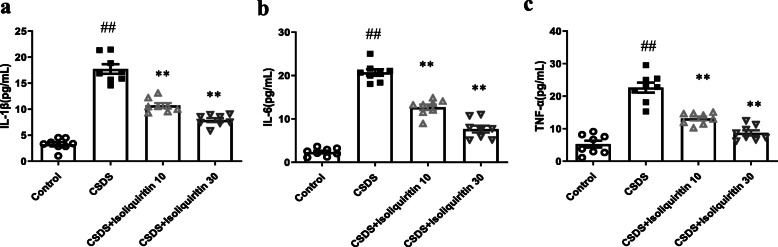
Effects of isoliquiritin on concentration of IL-1β, IL-6, and TNF-α in the hippocampus of CSDS mice. **a** IL-1β, **b** IL-6, and **c** TNF-α. Data are presented as mean ± SEM (*n* = 8 per group). ^##^*p* < 0.01, compared to control group; **p < 0.01, compared to LPS group

We next evaluated the effect of isoliquiritin on inflammation response applying Western blot and ELISA assays. As shown in Fig. 8e–h, chronical social defeat stress resulted in elevated protein levels of NLRP3 (*F* (3, 8) = 51.87, *p* < 0.0001), cleaved Caspase-1 (*F* (3, 8) = 21.34, *p* = 0.0004), IL-1β (*F* (3, 8) = 48.51, *p* < 0.0001), and GSDMD-N (*F* (3, 8) = 35.95, *p* < 0.0001) in Western blot assessment. Increased serum concentration of IL-1β (*F* (3, 28) = 108.4, *p* < 0.0001), IL-6 (*F* (3, 28) = 138.0, *p* < 0.0001) and TNF-α (*F* (3, 28) = 49.49, *p* < 0.0001) were also found in CSDS-challenged mice (Fig. 9), while these effects were completely reversed by isoliquiritin 30 mg/kg and partially by isoliquiritin 10 mg/kg, suggesting that treatment with the isoliquiritin significantly ameliorated inflammation response in CSDS mice.

#### Isoliquiritin affected neuron death

We detected the survival and alternation of neuronal cells, DNA fragmentation and NeuN expression to determine if pyroptotic induced cell death was blocked by isoliquiritin administration.

**Fig. 10:**
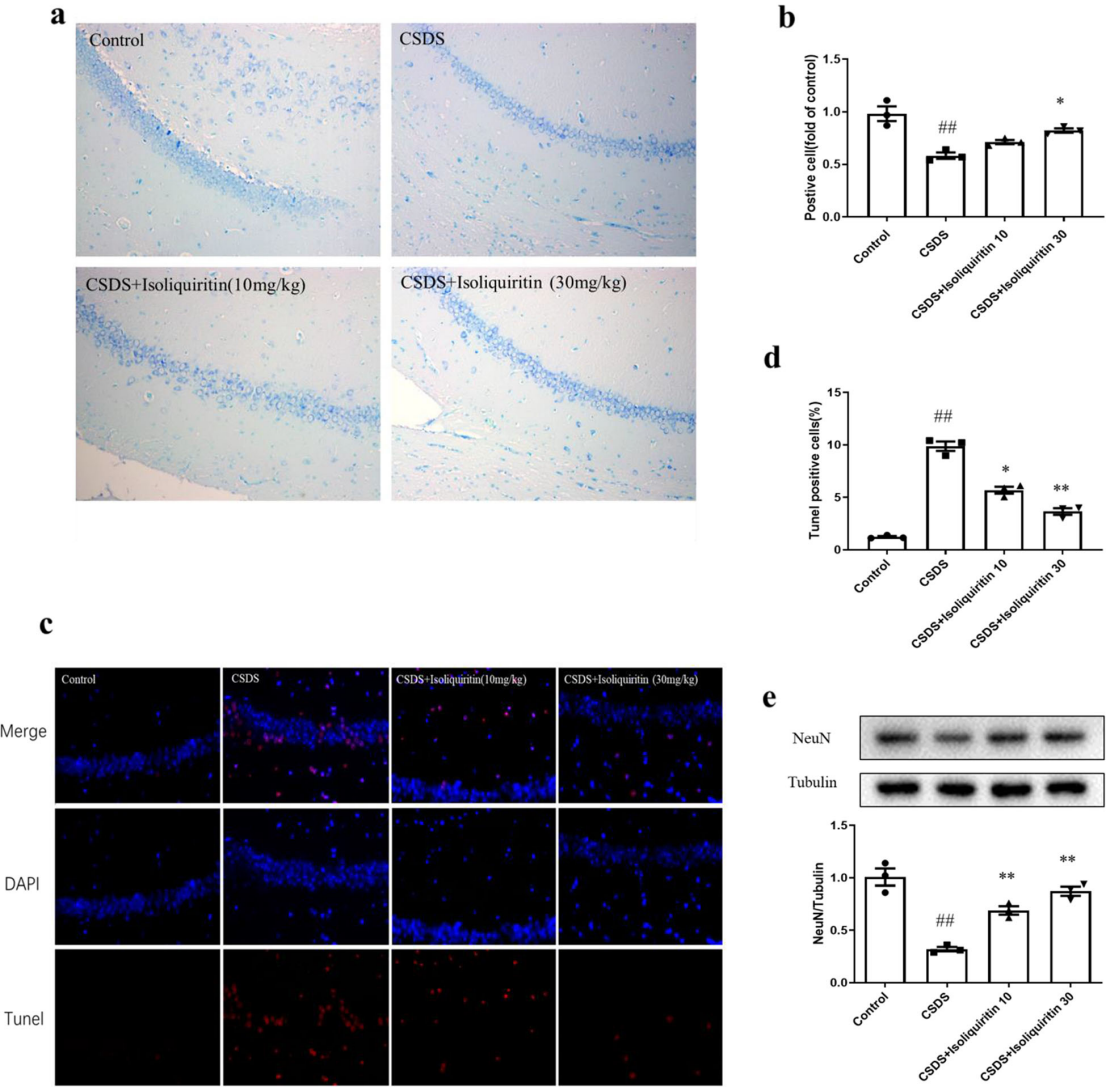
Influence of isoliquiritin on pyroptosis-related neuronal death in the hippocampus of CSDS mice. **a**, **b** Nissl staining of survived neurons. **c**, **d** TUNEL assay of neuron death. **e** NeuN protein expression in Western blot. Data are presented as mean ± SEM (*n* = 3 per group). ^##^*p* < 0.01, compared to control group; *p < 0.05, **p < 0.01, compared to LPS group

In Nissl staining, the neurons in the hippocampus were clear and intact in control group (Fig. 10a, b). Whereas in CSDS model group, the number of Nissl-positive cells was noticeably decreased (*F* (3, 8) = 17.58, *p* = 0.0007), and impaired neurons increased, which had irregular neuronal cell bodies, shrinking and hyperchromatic nuclei. Interestingly, these effects were clearly mitigated by isoliquiritin intervention (30 mg/kg).

The results in TUNEL staining demonstrated that the model group exhibited an evident increase in TUNEL-positive cells (*F* (3, 8) = 127.2, *p* < 0.0001) compared with the control group (Fig. 10c, d). However, the apoptotic cell number markedly declined after treatment with isoliquiritin (10 mg/kg; 30 mg/kg).

The protein level of NeuN was analyzed using Western blot (Fig. 10e). Declined NeuN level was observed in CSDS mice (*F* (3, 8) = 33.57, *p* < 0.0001), which was restored by isoliquiritin treatment (10 mg/kg; 30 mg/kg).

### miRNA-27a inhibitor compromised isoliquiritin-generated therapeutic efficacy in CSDS mice

#### miRNA-27a inhibitor blocked isoliquiritin-generated efficacy on depressive behaviors

**Fig. 11:**
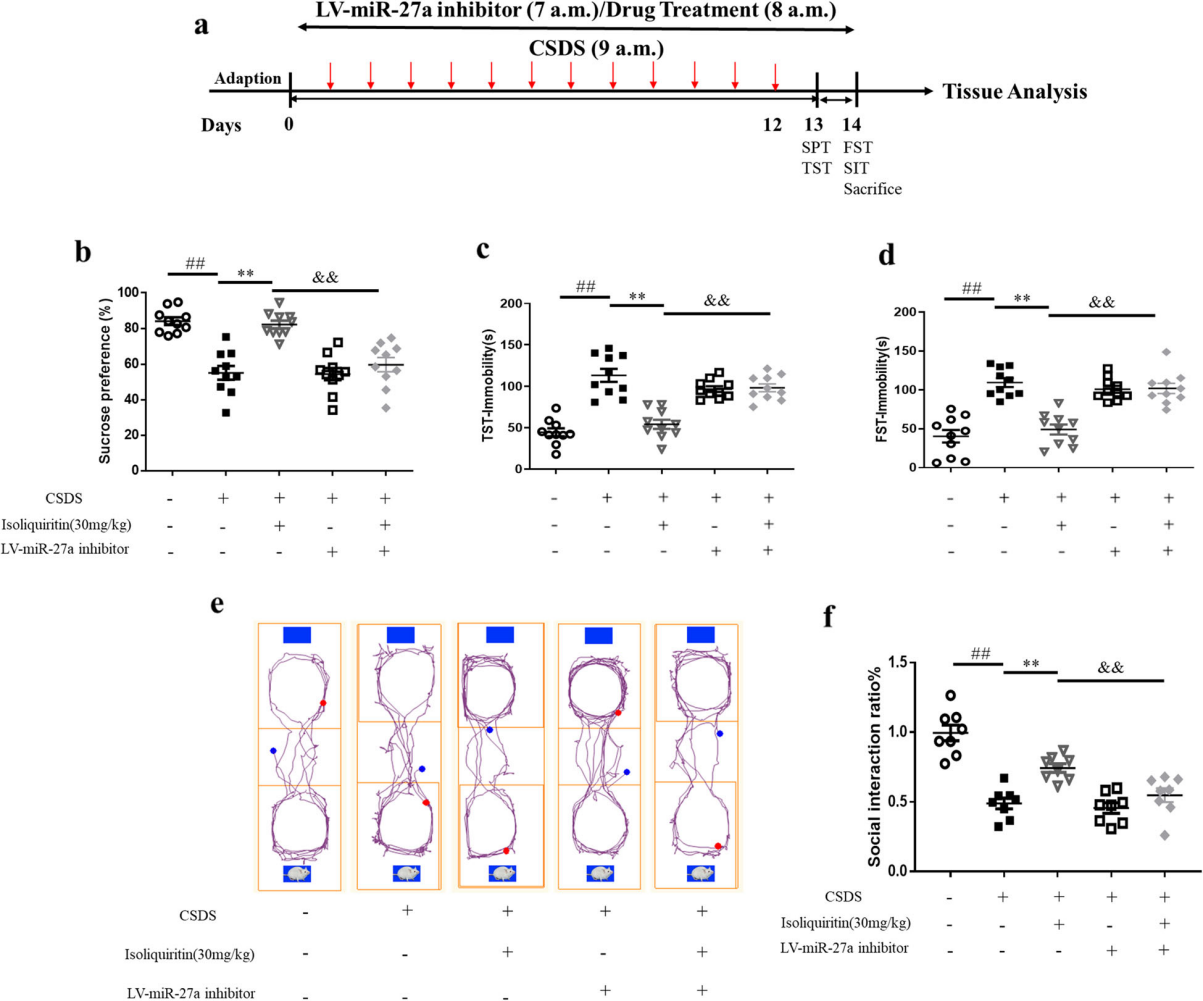
Impact of miRNA-27a inhibitor on isoliquiritin-generated antidepressant and anxiolytic efficacy in CSDS mice. **a** Schematic timeline of the experimental procedure. **b** Sucrose preference test (*n* = 13 per group). **c** Tail suspension test (*n* = 10 per group). **d** Forced swimming test (*n* = 10 per group). **e**, **f** Social interaction test (*n* = 8 per group). Data are presented as mean ± SEM. ^##^*p* < 0.01, compared to blank group; **p < 0.01, compared to CSDS group; ^&^*p* < 0.05, ^&&^*p* < 0.01, compared to CSDS+ isoliquiritin group

To elucidate the mechanism of miRNA-27a in regulating isoliquiritin-related antidepressant efficacy, lentivirus miRNA-27a inhibitor was used in this study (Fig. 11a). Clearly, treatment with isoliquiritin (30 mg/kg) increased the sucrose preference in SPT (*F* (1, 36) = 22.38, *p* < 0.0001) and social interaction in SIT (*F* (1, 28) = 18.85, *p* = 0.0002), and shortened the immobility duration in both TST (*F* (1, 36) = 26.42, *p* < 0.0001) and FST (*F* (1, 36) = 25.70, *p* < 0.0001) tests on CSDS model (Fig. 11b–f). However, pretreatment with miRNA-27a inhibitor significantly reversed isoliquiritin-generated beneficial effects in SPT (*F* (1, 36) = 11.61, *p* = 0.0016), SIT (*F* (1, 28) = 8.193, *p* = 0.0079), TST (*F* (1, 36) = 6.083, *p* = 0.0185), and FST (*F* (1, 36) = 14.35, *p* = 0.0006) assessments. These data demonstrated that activation of miRNA-27a is required for the antidepressant actions of isoliquiritin.

#### miRNA-27a inhibitor abolished isoliquiritin-generated effects on miRNA-27a-regulated pyroptosis signaling

**Fig. 12:**
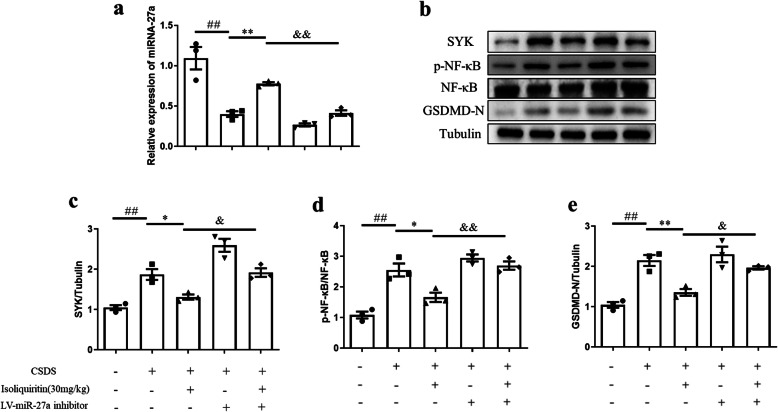
Impact of miRNA-27a inhibitor on isoliquiritin-generated deactivation of pyroptosis signaling in the hippocampus of CSDS mice. **a** mRNA expression level of miRNA-27a. **b** Representative Western blots. **c**–**e** Protein levels of SYK, p-NF-κB, and GSDMD-N. Data are presented as mean ± SEM (*n* = 3 per group). ^##^*p* < 0.01, compared to blank group; *p < 0.05, **p < 0.01, compared to CSDS group; ^&^*p* < 0.05, ^&&^*p* < 0.01, compared to CSDS+ isoliquiritin group

We also explored whether miRNA-27a is required for isoliquiritin-generated stimulation of miRNA-27a-regulated pyroptosis pathway in the hippocampus (Fig. 12). In the hippocampus of CSDS mice, treatment with isoliquiritin (30 mg/kg) elicited a significant promotion in the mRNA expression of miRNA-27a (*F* (1, 8) = 47.11, *p* = 0.0001), and obvious decrease in the protein levels of SYK (*F* (1, 8) = 25.72, *p* = 0.0010), p-NF-κB (*F* (1, 8) = 13.09, *p* = 0.0068), and pyroptosis executor GSDMD-N, which were all ablated by miRNA-27a inhibitor (miRNA-27a, *F* (1, 8) = 30.26, *p* = 0.0006; SYK, *F* (1, 8) = 29.75, *p* = 0.0006; p-NF-κB, *F* (1, 8) = 20.48, *p* = 0.0019). These results support the conclusion that miRNA-27a activation is necessary for isoliquiritin-induced restoration in miRNA-27a mediated pyroptosis pathway.

#### miRNA-27a inhibitor compromised isoliquiritin-generated effects on pyroptosis-related neuron death

**Fig. 13:**
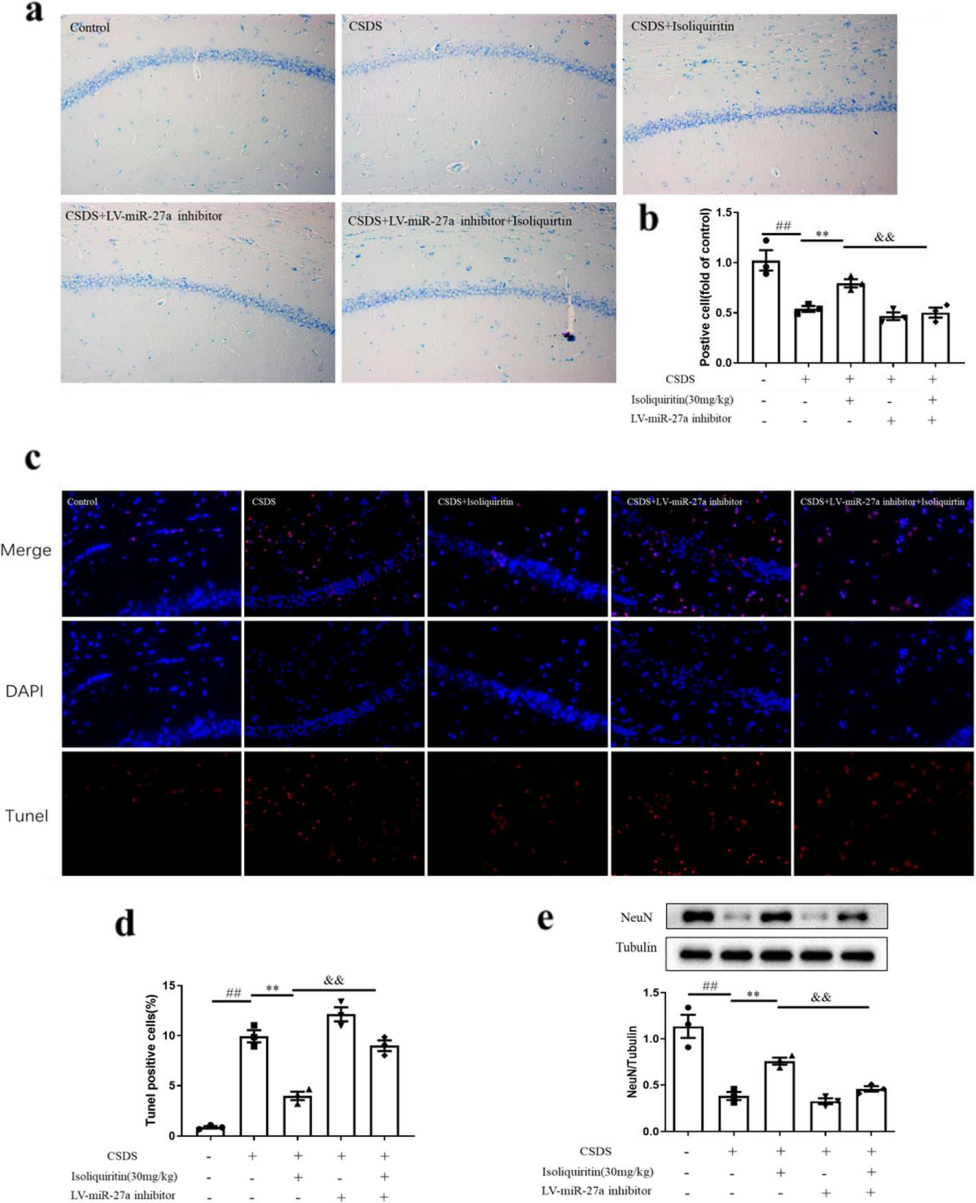
Influence of miRNA-27a inhibitor on isoliquiritin-generated decrease of neuronal death in the hippocampus of CSDS mice. **a**, **b** Nissl staining of survived neurons. **c**, **d** TUNEL assay of neuron death. **e** NeuN protein expression in Western blot. Data are presented as mean ± SEM (*n* = 3 per group). ^##^*p* < 0.01, compared to blank group; **p < 0.01, compared to CSDS group; ^&&^*p* < 0.01, compared to CSDS+ isoliquiritin group

To further investigate the impact of miRNA-27a on isoliquiritin-induced attenuation of pyroptosis-related neuron death, Nissl staining, TUNEL assay, and Western blot were carried out (Fig. 13). Nissl staining and TUNEL assay results indicated that isoliquiritin (30 mg/kg) improved the survival and morphology of neurons (*F* (1, 8) = 13.37, *p* = 0.0064), as well as pyroptosis-induced cell death (*F* (1, 8) = 62.35, *p* < 0.0001) in CSDS mice. In Western blot assay, the upregulated NeuN Protein expression was observed in CSDS mice after isoliquiritin treatment (30 mg/kg). Nonetheless, these effects were all abolished by miRNA-27a inhibitor (Nissl, *F* (1, 8) = 20.90, *p* = 0.0018; TUNEL, *F* (1, 8) = 39.17, *p* = 0.0002). These findings suggested that isoliquiritin-related efficacy on pyroptosis was dependent on miRNA-27a activation.

### Role of miRNA-27a in the effect of isoliquiritin in alleviating LPS and ATP induced neuroinflammation in vitro

**Fig. 14:**
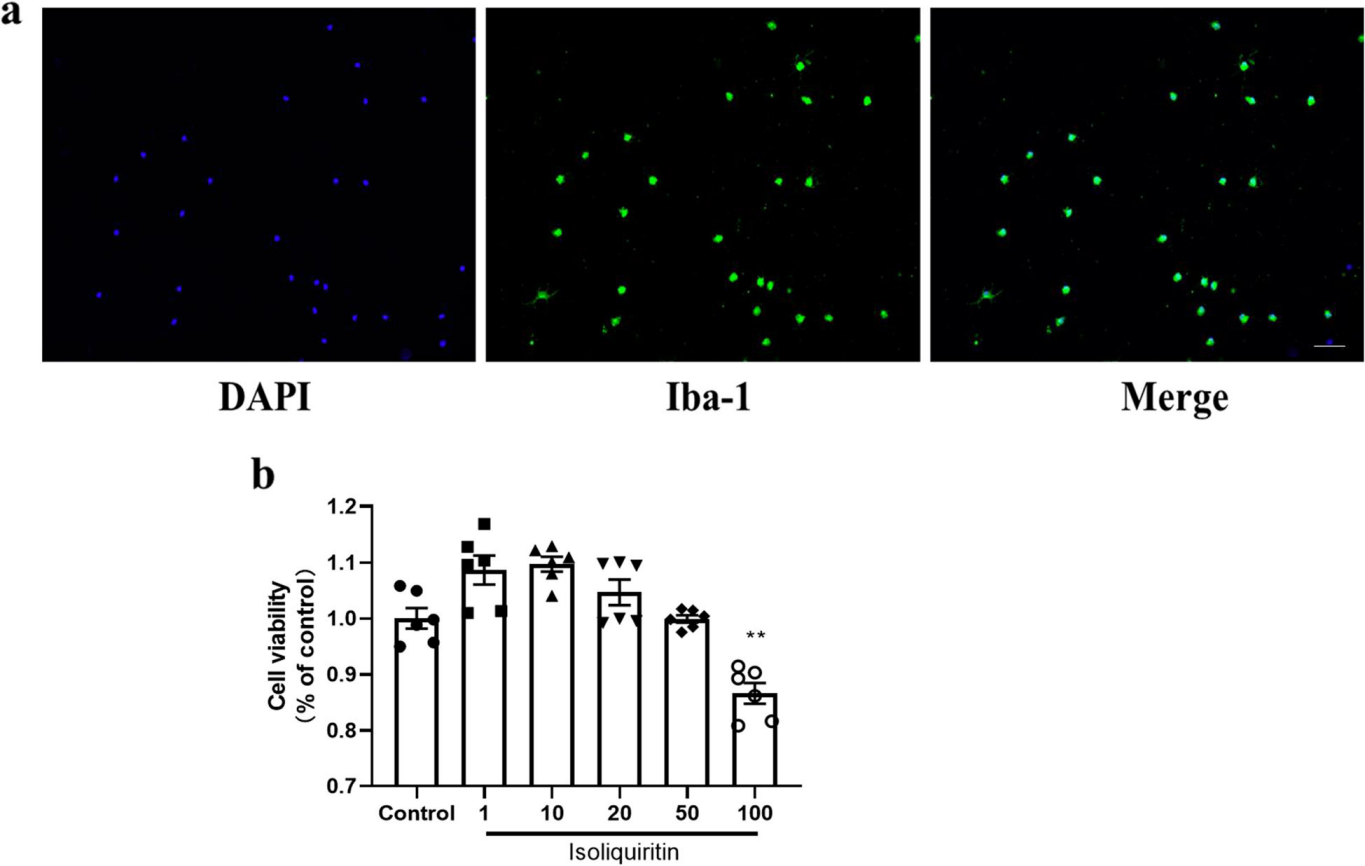
Details of primary mouse microglial cell culture and cell viability following isoliquiritin treatment. **a** Iba-1 immunostaining images of primary microglia isolated using shaking. Scale bar = 50 μm. **b** Cell viability in MTT assay. Data are presented as mean ± SEM (*n* = 6 per group). **p < 0.01, compared to control group

Next, we examined the effect of isoliquiritin on NLRP3 inflammasome activation in vitro by stimulating primary microglia cells with LPS and ATP (Fig. 14a).

Preliminary experiment was performed in order to measure the toxicity of isoliquiritin using MTT assay (Fig. 14b). The result showed that the maximum concentration that had no effect on reducing cell viability was 50 μM for isoliquiritin (*F* (5, 30) = 20.21, *p* < 0.0001). Thus, this dose was used for future experiments.

**Fig. 15:**
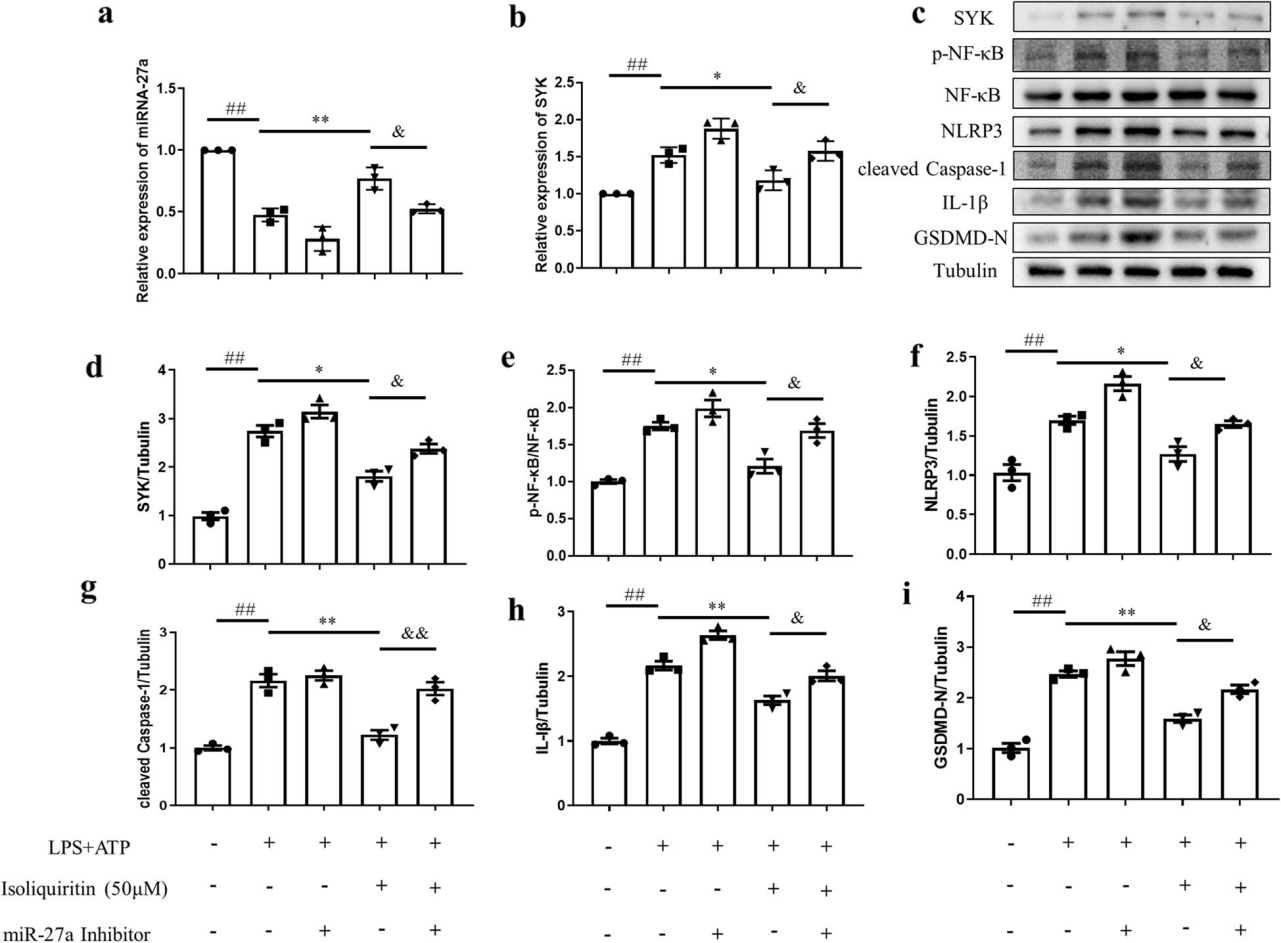
Effect of miRNA-27a inhibitor on isoliquiritin-generated cytoprotective effects in LPS+ATP-treated primary microglia cells. **a**, **b** mRNA levels of miRNA-27a and SYK. **c** Representative Western blots. **d**–**i** Protein expressions of SYK, p-NF-κB, NLRP3, cleaved Caspase-1, IL-1β, and GSDMD-N. Data are presented as mean ± SEM (*n* = 3 per group). ^##^*p* < 0.01, compared to blank group; *p < 0.05, **p < 0.01, compared to LPS+ATP group; ^&^*p* < 0.05, ^&&^*p* < 0.01, compared to LPS+ATP+Isoliquiritin group

Our data suggested that LPS and ATP treatment significantly decreased miRNA-27a mRNA expression and elevated SYK mRNA level, and upregulated the protein expressions of SYK, p-NF-κB, NLRP3, cleaved Caspase-1, IL-1β, and GSDMD-N in primary microglia (*p* < 0.05, Fig. 15). Nonetheless, pretreatment with isoliquiritin rescued the abnormality in miRNA-27a (*F* (1, 8) = 39.67, *p* = 0.0002) and SYK (*F* (1, 8) = 18.84, *p* = 0.0025) mRNA levels, and restored the proteins levels of SYK (*F* (1, 8) = 54.28, *p* < 0.0001), p-NF-κB (*F* (1, 8) = 20.92, *p* = 0.0018), NLRP3 (*F* (1, 8) = 41.58, *p* = 0.0002), cleaved Caspase-1 (*F* (1, 8) = 34.44, *p* = 0.0004), IL-1β (*F* (1, 8) = 68.30, *p* < 0.0001), and GSDMD-N (*F* (1, 8) = 63.86, *p* < 0.0001). Interestingly, the protective effects of isoliquiritin on miRNA-27a (*F* (1, 8) = 26.34, *p* = 0.0009) and SYK (*F* (1, 8) = 25.92, *p* = 0.0009) mRNA expressions as well as protein levels of SYK (*F* (1, 8) = 17.84, *p* = 0.0029), p-NF-κB (*F* (1, 8) = 15.38, *p* = 0.0044), NLRP3 (*F* (1, 8) = 33.34, *p* = 0.0004), cleaved Caspase-1(*F* (1, 8) = 19.94, *p* = 0.0021), IL-1β (*F* (1, 8) = 36.15, *p* = 0.0003), and GSDMD-N (*F* (1, 8) = 22.50, *p* = 0.0015) were all reversed by miRNA-27a inhibitor.

**Fig. 16:**
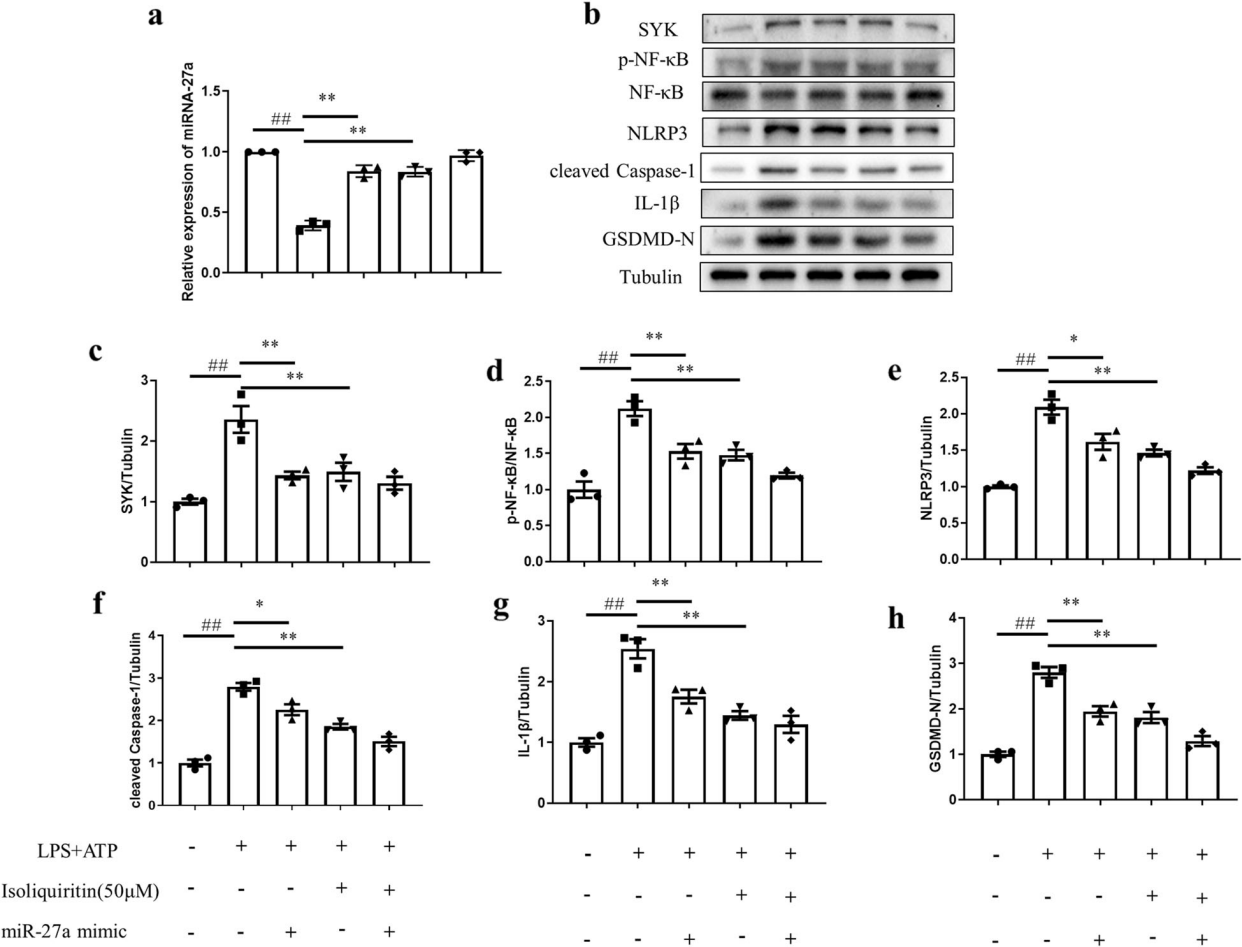
Effect of miRNA-27a mimics and isoliquiritin in LPS+ATP-treated primary microglia cells. **a** mRNA expression of miRNA-27a. **b** Representative Western blots. **c**–**h** Protein levels of SYK, p-NF-κB, NLRP3, cleaved Caspase-1, IL-1β, and GSDMD-N. Data are presented as mean ± SEM (*n* = 3 per group). ^##^*p* < 0.01, compared to blank group; *p < 0.05, **p < 0.01, compared to LPS+ATP group

Moreover, Fig. 16 revealed that isoliquiritin ameliorated LPS and ATP induced decline in miRNA-27a mRNA expression (*F* (1, 8) = 50.92, *p* < 0.0001) and increase in proteins levels of SYK (*F* (1, 8) = 24.70, *p* = 0.0011), p-NF-κB (*F* (1, 8) = 34.79, *p* = 0.0004), NLRP3(*F* (1, 8) = 39.02, *p* = 0.0002), cleaved Caspase-1 (*F* (1, 8) = 69.14, *p* < 0.0001), IL-1β (*F* (1, 8) = 38.29, *p* = 0.0003), and GSDMD-N (*F* (1, 8) = 50.13, *p* = 0.0001), which was similar to the effects elicited by miRNA-27a mimics.

## Discussion

**Fig. 17:**
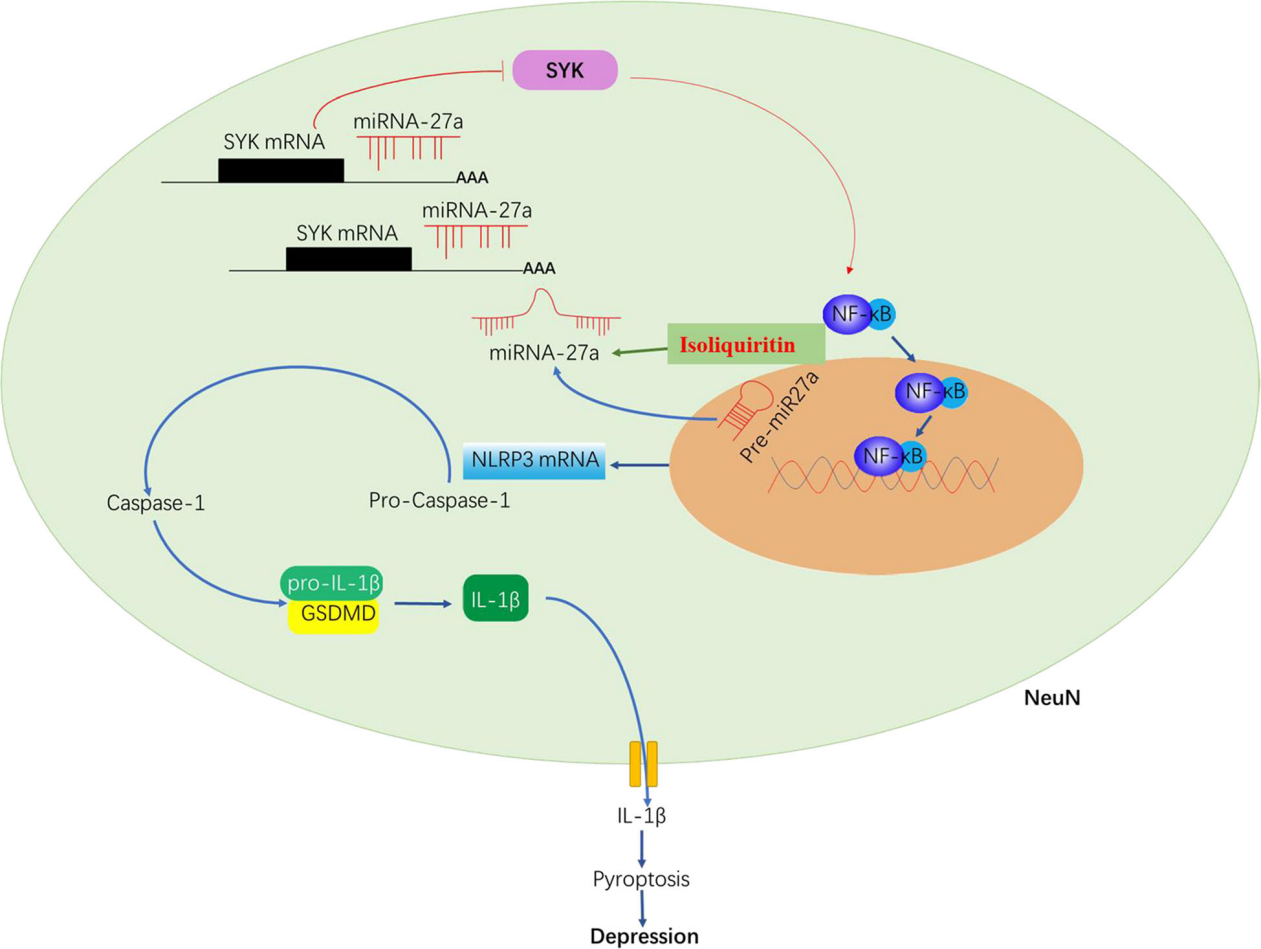
A hypothetical scheme of the molecular mechanisms underlying isoliquiritin-related antidepressant efficacy

In this study, we explored the antidepressant property of isoliquiritin and its potential mechanism (Fig. 17). Our results showed that isoliquiritin successfully attenuated LPS- or CSDS-induced depressive symptoms and CSDS-elicited anxiety behaviors. In the hippocampus, expression levels of miRNA-27a/SYK/NF-κB cascade and NLRP3-mediated inflammation response and pyroptosis were all improved after isoliquiritin administration. Furthermore, isoliquiritin protected primary microglia against LPS and ATP induced NLRP3 inflammasome activation in vitro, evidenced by declined protein levels of p-NF-κB, NLRP3, cleaved Caspase-1, IL-1β, and GSDMD-N, promoted miRNA-27a mRNA expression and reduced the mRNA and protein levels of SYK. However, miRNA-27a inhibitor significantly reversed isoliquiritin-generated therapeutic efficacy in CSDS mice and in vitro. Moreover, the cytoprotective effect of isoliquiritin was similar to that of miRNA-27a mimics in LPS and ATP-treated primary microglia.

LPS depression model is widely used to study inflammation-associated depression. Lipopolysaccharide (LPS) is a component of the cell membrane of Gram-negative bacteria, which provokes the production of proinflammatory cytokines in brain and periphery [25]. In rodents, LPS triggered the development of sickness behaviors such as decrease in body weight, food intake, and locomotor activity that resolves after 14–18 h, followed by a phase of depression-like behaviors including declined sucrose preference rate and prolonged immobility duration in TST and FST [26, 27].

Chronic social defeat stress (CSDS) paradigm has been widely used to explore the mechanisms underlying the pathogenesis of depression- and anxiety-related behaviors [28]. In real-life situations, people frequently encounter stimuli resulted from the interaction with other people, and social challenge appears to be one of the most prevalent stressors in humans and social animals [29]. Repeated exposures to social defeat stress in rodents result in significant anhedonia, behavioral despair, social avoidance, and heightened anxiety [30]. In our study, mice experienced LPS or CSDS displayed decreased sucrose preference rate and social interaction ratio, and prolonged immobility in TST and FST, suggesting the development of depression after LPS or CSDS challenges. However, isoliquiritin administration profoundly improved the behavior defects in SPT, TST, FST, and SIT, indicating that isoliquiritin was effective in alleviating depression in mice. In previous study, isoliquiritin was shown to mitigate behavioral despair in normal mice [14]. Here, we further demonstrated that isoliquiritin could confer antidepressant-like activity in the rodent models of depression. Additionally, existing evidence suggested that CSDS mice displayed heightened anxiety in OFT test, which is consistent with our result [31]. Nonetheless, treatment with isoliquiritin evidently increased time spent in center in OFT, indicative of an anxiolytic effect of isoliquiritin.

NLRP3 is one of the most extensively studied NOD-like receptors, which play essential roles in triggering inflammasome-mediated neuroinflammation in microglia. The activation of NLRP3 promotes the secretion of Caspase-1, which increases the cleavage of IL-1β and GSDMD, and then induces pyroptosis and inflammation response. The NLRP3 cascade has been implicated in multiple diseases, such as Alzheimer’s disease, Parkinson’s disease, and stroke. Gene expression analysis revealed that the expression levels of NLRP3, Caspase-1, and IL-1β were elevated in the cultured PBMCs from AD patients [32]. Enhanced IL-1β expression and Caspase-1 activity were detected in the serum samples of PD patients, and increased levels of NLRP3, Caspase-1, and IL-1β were found in the midbrain of PD mice model [33]. NLRP3-inflammasome inhibitor MCC950 suppressed the activation of Caspase-1 and IL-1β, leading to reduction of infarction, edema, and Hb content as well as improved neurological deficits in transient middle cerebral artery occlusion mice model [34].

The NLRP3 cascade is also involved in depression. Increased NLRP3 and IL-1β activity were observed in the prefrontal cortex of depressed rats [35]. Melatonin, a hormone produced from L-tryptophan, remarkably enhanced LPS-induced depressive symptoms in FST and TST assessments, which was linked to the deactivation of NLRP3 controlled inflammation response and pyroptosis [5]. Postmortem studies demonstrated that the protein expression and mRNA levels of IL-1β, IL-6, and TNF-α were remarkably increased in the prefrontal cortex of depressed subjects who died by suicide [36]. In our study, mice underwent LPS or CSDS challenge displayed elevated levels of NLRP3, Caspase-1, IL-1β, IL-6, GSDMD, and TNF-α; decreased neuron survival; and increased neuronal cell death, which were all attenuated by isoliquiritin intervention. These results confirming the essential role of NLRP3-mediated pyroptosis cascade in the development of depression and Isoliquiritin-related effects on depressive behaviors.

miRNA-27a is a member of the miRNA family, which modulates gene expression of various biological processes including immune response [37]. miRNA-27a was documented to directly target SYK and then stimulate the NF-κB signal [7, 8]. In this study, the expression of the SYK gene was significantly decreased after miRNA-27a mimic incubation, confirming that regulation function of miRNA-27a on SYK. The miRNA-27a/SYK/NF-κB axis has been proposed to regulate the NLRP3 cascade. For instance, overexpression of miRNA-27a was able to suppress IL-1β-induced inflammatory response in chondrocytes [38]. SYK inhibitor R406 significantly deactivated NLRP3, Caspase-1, and GSDMD-N signaling; reduced the concentration of IL-1β; and attenuated GSDMD-N induced membrane pores in mice with ischemic stroke [39]. Ju et al. discovered that LPS treatment obviously increased the levels of proinflammatory cytokines including TNF-α, IL-1β, and IL-6 in the BAL fluid of mice that received LPS injection, and promoted the number of TUNEL-positive cells in the lung, while agomir-27a intervention greatly alleviated LPS-induced increase of proinflammatory cytokines and cell death [40].

The miRNA-27a/SYK/NF-κB axis was also involved in depression. Postmortem brain studies revealed that the miRNA-27a expression was downregulated in the prefrontal cortex of depressed suicide subject [41]. Depressed rats exhibited decreased levels of miRNA-27a in the hippocampus and peripheral blood [42]. Honokiol, a bioactive polyphenolic substance, significantly shortened the immobility duration of LPS mice in both TST and FST experiments, which was strongly related to decreased NF-κB activity in the hippocampus [43]. Our work discovered that the miRNA-27a expression was reduced in the serum of depressed patient and rodent animals. Meanwhile, mice experienced LPS or CSDS modeling displayed reduced levels of miRNA-27a, SYK, and NF-κB. These results support the critical role of the miRNA-27a/SYK/NF-κB axis in the development of depression. In addition, treatment with isoliquiritin remarkably improved the depressive behaviors in LPS and CSDS mice and attenuated NLRP3-mediated pyroptosis and inflammation response. In vitro study showed that similar to miRNA-27a mimics, isoliquiritin enhanced miRNA-27a expression and declined SYK level in LPS and ATP-stimulated primary microglia, and downregulated protein expressions of p-NF-κB, NLRP3, cleaved Caspase-1, IL-1β, and GSDMD-N. These findings confirmed the regulation role of miRNA-27a/SYK/NF-κB axis in the NLRP3 cascade activation and isoliquiritin-induced antidepressant activities. Furthermore, after treatment with miRNA-27a inhibitor, isoliquiritin-induced therapeutic efficacy was reversed in CSDS mice and in vitro, reflecting the requirement of miRNA-27a activation in isoliquiritin-related antidepressant activity, and further highlighting the critical role of miRNA-27a in the treatment of depression.

## Conclusions

In summary, our findings indicated that isoliquiritin could significantly mitigate depressive symptoms in mice, which was dependent on miRNA-27a/SYK/NF-κB axis-regulated suppression of pyroptosis via NLRP3 cascade. The present study revealed the antidepressant property isoliquiritin and its underlying mechanism and provided novel therapeutic strategies for the treatment of depression.

## Supplementary Information

**Additional file 1: Figure S1.** miRNA-27a mRNA expression in different genders of depressed patients. Data are presented as mean ± SEM (*n*=12 per group). Figure S2. Influence of Isoliquiritin and Fluoxetine on naïve animals. Male C57BL6/J mice were administrated with Isoliquiritin (30mg/kg) or Fluoxetine (20mg/kg) for 14 days, then behavioral tests and ELISA assay were carried out. (a) Tail suspension test, (b) Forced swimming test, (c) Sucrose preference test, (d) TNF-α, (e) IL-1β, (f) IL-6. Data are presented as mean ± SEM (*n*=10 per group).

## Data Availability

All data generated or analyzed during this study are included in this published article.

## Abbreviations

LPS: Lipopolysaccharide
ATP: Adenosine triphosphate
CSDS: Chronic social defeat stress
GSDMD: Gasdermin D
SSRIs: Selective serotonin reuptake inhibitors
NLRs: NOD-like receptors
CSF: Cerebral spinal fluid
miRNA: MicroRNA
SYK: Spleen tyrosine kinase
TST: Tail suspension test
FST: Forced swimming test
SPT: Sucrose preference test
OFT: Open field test
SIT: Social interaction test
qRT-PCR: Quantitative real-time polymerase chain reaction
WT: Wild-type
MUT: Mutant
PVDF: Polyvinylidene difluoride
ELISA: Enzyme-linked immunosorbent assay
ANOVA: Analysis of variance
