# Supplementary material for: Isoliquiritin ameliorates depression by suppressing NLRP3-mediated pyroptosis via miRNA-27a/SYK/NF-κB axis
Source: J Neuroinflammation. 2021 Jan 5;18:1. doi: 10.1186/s12974-020-02040-8 (PMC7786465; doi:10.1186/s12974-020-02040-8)
Supplement: Supplementary file 1 — Additional file 1: Figure S1. miRNA-27a mRNA expression in different genders of depressed patients. Data are presented as mean ± SEM (n=12 per group). Figure S2. Influence of Isoliquiritin and Fluoxetine on naïve animals. Male C57BL6/J mice were administrated with Isoliquiritin (30mg/kg) or Fluoxetine (20mg/kg) for 14 days, then behavioral tests and ELISA assay were carried out. (a) Tail suspension test, (b) Forced swimming test, (c) Sucrose preference test, (d) TNF-α, (e) IL-1β, (f) IL-6. Data are presented as mean ± SEM (n=10 per group). [file 12974_2020_2040_MOESM1_ESM.docx]

**Supplemental Material**

**Figure S1.** miRNA-27a mRNA expression in different genders of depressed patients.

Data are presented as mean ± SEM (n=12 per group).

**Figure S****2.** Influence of Isoliquiritin and Fluoxetine on naïve animals.

Male C57BL6/J mice were administrated with Isoliquiritin (30mg/kg) or Fluoxetine (20mg/kg) for 14 days, then behavioral tests and ELISA assay were carried out. (a) Tail suspension test, (b) Forced swimming test, (c) Sucrose preference test, (d) TNF-α, (e) IL-1β, (f) IL-6. Data are presented as mean ± SEM (n=10 per group).

**Figure S1**


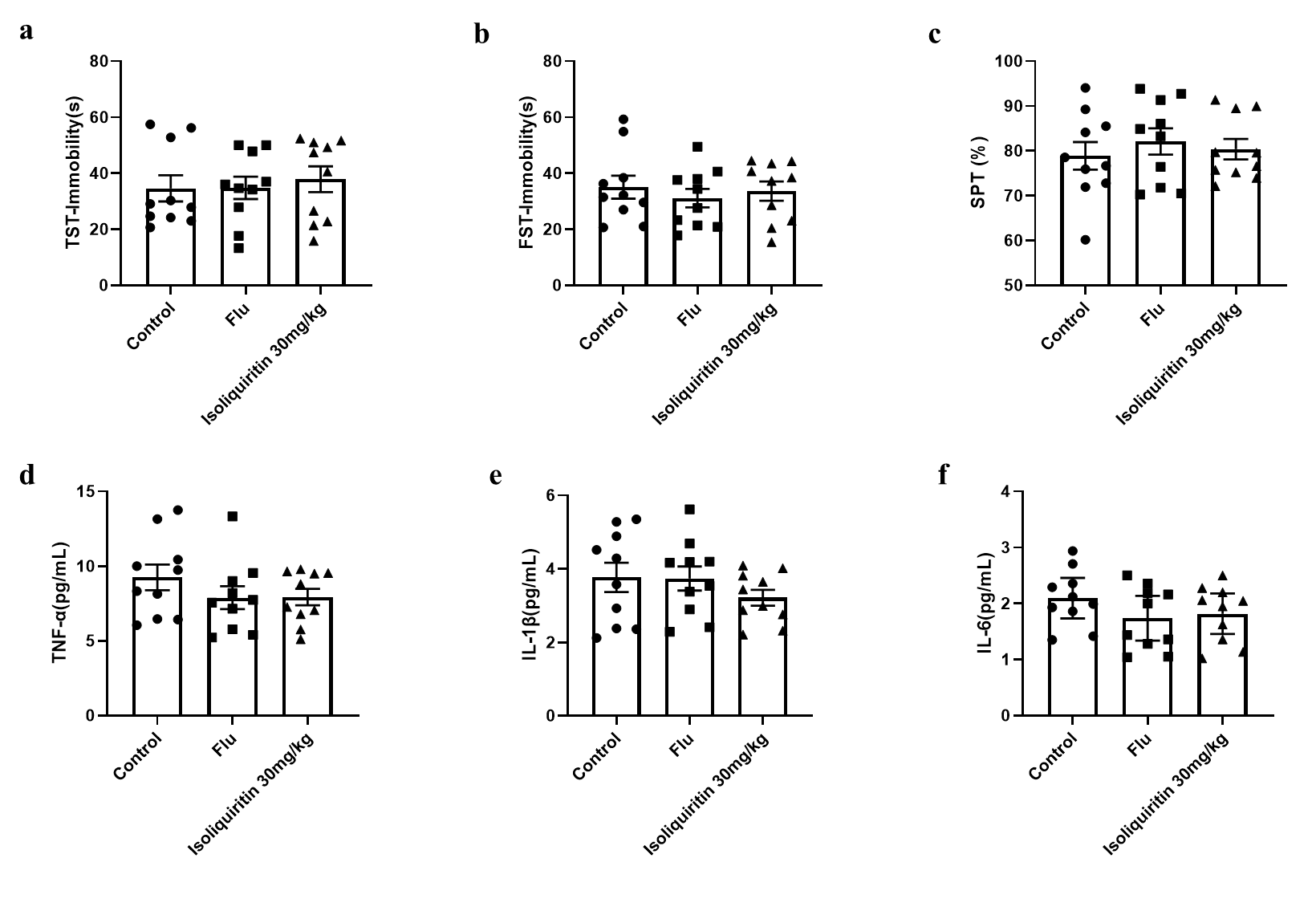


**Figure S2**
